# Detection and Identification of Allergens from Canadian Mustard Varieties of *Sinapis alba* and *Brassica juncea*

**DOI:** 10.3390/biom9090489

**Published:** 2019-09-14

**Authors:** Lamia L’Hocine, Mélanie Pitre, Allaoua Achouri

**Affiliations:** Saint-Hyacinthe Research and Development Centre, Agriculture and Agri-Food Canada, 3600 Casavant Blvd. W. Saint-Hyacinthe, QC J2S 8E3, Canada

**Keywords:** mustard, *Sinapis alba*, *Brassica juncea*, allergens, IgE-binding, protein extraction, mass spectrometry, immunoblotting, sequence alignment

## Abstract

Currently, information on the allergens profiles of different mustard varieties is rather scarce. Therefore, the objective of this study was to assess protein profiles and immunoglobulin E (IgE)-binding patterns of selected Canadian mustard varieties. Optimization of a non-denaturing protein extraction from the seeds of selected mustard varieties was first undertaken, and the various extracts were quantitatively and qualitatively analyzed by means of protein recovery determination and protein profiling. The IgE-binding patterns of selected mustard seeds extracts were assessed by immunoblotting using sera from mustard sensitized and allergic individuals. In addition to the known mustard allergens—Sin a 2 (11S globulins), Sin a 1, and Bra j 1 (2S albumins)—the presence of other new IgE-binding protein bands was revealed from both *Sinapis alba* and *Brassica juncea* varieties. Mass spectrometry (MS) analysis of the in-gel digested IgE-reactive bands identified the unknown ones as being oleosin, β-glucosidase, enolase, and glutathione-S transferase proteins. A bioinformatic comparison of the amino acid sequence of the new IgE-binding mustard proteins with those of know allergens revealed a number of strong homologies that are highly relevant for potential allergic cross-reactivity. Moreover, it was found that Sin a 1, Bra j 1, and cruciferin polypeptides exhibited a stronger IgE reactivity under non-reducing conditions in comparison to reducing conditions, demonstrating the recognition of conformational epitopes. These results further support the utilization of non-denaturing extraction and analysis conditions, as denaturing conditions may lead to failure in the detection of important immunoreactive epitopes.

## 1. Introduction

Mustard is one of the priority food allergens regulated by Canada, the European Union, and the Gulf Cooperation Council (GCC), including the countries of Saudi Arabia, United Arab Emirates (UAE), Kuwait, Bahrain, Oman, Qatar, and Yemen. The inclusion of mustard on the regulatory allergen list of these countries was based on the view that mustard allergy poses a serious problem because of its widespread use and high allergenic potency [1,2]. There are little data available on the prevalence rates of mustard allergy, but it seems to vary around the world and appears to be more common in Europe, accounting for 1–7% of food allergy based on estimated prevalence in France [3,4]. Mustard allergy is also well documented in a number of published clinical studies reporting on severe systemic reactions, including anaphylaxis following exposure to very small amounts of mustard [5,6,7,8].

The international mustard market is led by Canada, which is the world’s second largest producer and the first exporter of mustard seed, holding a 57% share of the market [9]. Canada produces food grade mustard of three market classes, namely, yellow (*Sinapis alba*), oriental, and brown (*Brassica juncea*) mustard. The different types of mustard vary in physical appearance and use. Of these, yellow mustard seeds are larger, have higher protein (31.4%) and lower oil (30.4%) contents, and are milder in pungency in comparison to brown and oriental seeds [10], which contain 27.1% and 26.1% of protein and 38.5% and 42.4% of oil, respectively [11].

The major market for yellow mustard is the North American condiment industry, where it is commonly used to produce dry milled flour (fine powder from dehulled seeds) for salad dressings, mayonnaise, barbecue sauces, pickles and is also used as an excellent emulsifying agent and stabilizer for processed meats. On the other hand, the wet milled mustard is mainly used for mustard paste and the whole ground seeds as a seasoning. Brown mustard is primarily exported to Europe, where it is used to produce condiments and specialty mustard such as Dijon mustard. Oriental mustard is primarily grown for export to Asian countries, where it is used to produce condiments or spicy cooking oil. Beyond these uses, there is a growing interest in mustard components including protein, oil, and mucilage for a large spectrum of food, pharmaceutical, and industrial applications due to their nutritional, biological, and functional properties. As a result, mustard utilization is expected to increase in the future with an increasing risk of mustard being present as a hidden ingredient in many prepared or prepackaged products.

To date, only four major allergens from yellow mustard (*S. alba*) have been identified, namely: (a) Sin a 1, characterized as a seed storage protein napin and belonging to the 2S albumin family with a molecular weight (MW) of 14 kDa [12,13,14,15,16,17,18]; (b) Sin a 2, belonging to the seed storage 11S globulin with a MW of 51 kDa dissociated in two chains of 36 and 23 kDa [19,20]; (c) Sin a 3, a non-specific lipid transfer protein/nsLTP lipid transfer protein, 12 kDa [21]; and (d) Sin a 4, profilin, 13–14 kDa [21,22]. From the oriental mustard, Bra j 1, a seed storage protein from the 2S albumin family with a MW of approximately 16 kDa has been also identified as a major mustard allergen [23,24]. Previous studies revealed that Bra j 1 and Sin a 1 have a homologous epitope [23,24]. These findings imply that individuals known to be sensitive to one species of mustard are likely to show sensitivity to other species. However, information on mustard allergens from different mustard classes (types) and varieties remains limited, which represents a major obstacle to effective risk management and development of specific diagnostic tools and therapeutic approaches. The objective of this study was therefore to use an optimized, non-denaturing protein extraction for the assessment of protein profiles and Immunoglobulin E (IgE) binding patterns of major Canadian mustard varieties using sera from sensitized/allergic mustard individuals. This research allows the detection and the identification of new mustard allergens.

## 2. Materials and Methods

### 2.1. Preparation of Mustard Seed Flours

Seven different varieties of mustard were used in this study—two varieties of *Sinapis alba* (AC Pennant and Andante) and five varieties of Brassica juncea (Duchess, Centennial Brown, AC Vulcan, Cutlass, and Dahinda). All mustard seed samples were generously offered by Dr. Janitha Wanasundara of the Saskatoon Research and Development Centre of Agriculture and Agri-Food Canada (Saskatoon, SK, Canada). The seeds were frozen in liquid nitrogen, ground to a fine powder using an analytical mill (IKA A11, IKA, Staufen, Germany), and defatted with hexane (1:5 w/v) under constant magnetic stirring. The slurry was filtered using a Whatman No. 4 filter paper, and extractions were repeated three times. Defatted samples were dried overnight (~10–12 h) in a fume hood in order to remove all traces of residual solvent. Defatted flours were then homogenized for 30 s in a coffee grinder (Custom Grind Deluxe, Hamilton Beach, Washington, WA, USA) and stored in screw-capped plastic tubes at −80 °C until further use. Protein content in the defatted flour samples was determined by Dumas combustion (Leco FP-428, Leco Corporation, St Joseph, MI, USA). Percent of protein was calculated from protein nitrogen using a conversion factor of 6.25.

### 2.2. Optimization of Mustard Seed Protein Extraction

Extractions were conducted at various pH values in order to evaluate the protein solubilization and the extraction efficiency on mustard proteins from the defatted flours. A 3*7 full factorial experimental design (63 combinations) was used to study the effects of three different extraction buffers [phosphate-buffered saline (0.01 M, pH 7.4), borate-buffered saline (0.1 M, pH 8.45), and carbonate buffer (0.05 M, pH 9.6)] on protein recovery of mustard varieties. Minitab Statistical Software (version 16) (Minitab Inc., State College, PA, USA) was used to design the experiments.

Each extraction buffer was used to extract 0.5 g of defatted flour from each mustard variety at a protein/buffer ratio of 1:250 (*w*/*v*). All extractions were conducted in 50 mL centrifuge tubes under constant shaking at 45 rpm using a LabRoller Rotator (Labnet International, Woodbridge, NJ, USA) at room temperature for 1 h. The crude extracts were transferred in 70 mL centrifuge bottles and centrifuged in a Beckman JA-18 fixed angle rotor in a Beckman J2-21 centrifuge (Beckman Intruments, Brea, CA, USA) at 16,000× *g* for 30 min at 4 °C. The supernatant was passed on a filter paper (Whatman filter paper No. 4, Whatman International Ltd., Maidstone, UK) and further filtered on 0.45 µm filters. The pH of the extracts was measured at the beginning and the end of the extraction time to verify its stability. The protein concentration of the mustard extracts was determined using the Bradford protein assay [25]. Clarified extracts were transferred in 2 mL cryogenic vials and stored at −80 °C until use.

All protein extraction experiments were performed in duplicate, and the present results are the average values of four determinations (two experimental × two analytical replicates). Analysis of variance (ANOVA) was carried out using XLSTAT version 2012.4.01 to compare data obtained from different samples. Tukey multiple comparison was used to discriminate among the means of the variables when necessary. Differences at *p* ≤ 0.05 were considered significant.

### 2.3. Protein Electrophoresis

Mustard seed extracts normalized to equal amounts of protein (10 µg) were subjected to SDS-PAGE under reducing and non-reducing conditions using pre-cast Any KD TGX gels (Bio-Rad Laboratories, Hercules, CA, USA) according to Laemmli [26]. The soluble extracts were mixed with an equal volume of Laemmli sample buffer with 5% (*v*/*v*) of β-mercaptoethanol (β-ME) and boiled for 5 min. Alternatively, electrophoresis was performed under non-reducing conditions by omitting the addition of β-ME. The gels were run at a constant voltage of 150 V for 90 min using TGS buffer (25 mM Tris, 192 mM glycine, and 0.1% SDS) in a Criterion cell (Bio-Rad). A molecular weight standard (Precision Plus Protein Standard) was included on each gel. After electrophoresis, gels were stained with Coomassie Brilliant Blue. Images were acquired by scanning stained gels using an Image Scanner III (GE Healthcare, Salt Lake City, UT, USA) operated by LabScan 6.0 software (GE Healtcare). For image and densitometry analysis, the Image Quant TL 7.0 Software (GE Healtcare) was used.

### 2.4. Immunoblotting

Immunoblotting was carried out with human sera obtained from two different sources. Two sera named P1 and P2 were from mustard sensitized and self-declared allergic donors and were purchased from Plasma Lab International (Everett, WA, USA). A third serum named P3 was obtained from a clinically confirmed mustard allergic patient of the Sainte-Justine University Hospital Center (Montreal, QC, Canada). The three sera—P1, P2, and P3—showed a level of sensitization of Class III to mustard with specific IgE antibody levels equal to 5.76, 3.76, and 6.3 [kilo units of antibody per liter (kUA/L)], respectively, following measurement with the Pharmacia ImmunoCAP^®^ system. Control sera were obtained through Plasma Lab from patients with an allergic history to dust mites but without food allergy. The study was approved by the Sainte-Justine’s Hospital Ethics Committee and the Human Research Ethics Committee of Agriculture and Agri-Food Canada.

For western blots, 2 µg of carbonate buffer protein extract from each mustard variety were separated by SDS-PAGE (performed as described above); the separated proteins were then transferred on a polyvinylidene fluoride (PVDF) membrane using a Mini Trans-Blot electrophoretic transfer cell (Bio-Rad) at 100 V for 1 h at 4 °C according to Towbin [27]. The blotted membranes were subsequently blocked in 5% (*w*/*v*) skim milk powder in phosphate-buffered saline with 0.1% Tween-20 (PBS-T) for 1 h at room temperature. Membranes were then incubated overnight at 4 °C with 1:2 (*v*/*v*) dilutions of the three sera. IgE was detected by using a horseradish peroxidase (HRP) conjugated mouse anti-human antibody (clone B3102E8, Southern Biotech, Birmingham, AL, USA). Immunoreactive bands were visualized using amplified Opti-4CN reagents (Bio-Rad) following manufacturer’s recommendations. The immunoblots were scanned and analyzed as previously mentioned for the SDS-PAGE gels.

### 2.5. Indirect ELISA

High-binding 96-well microtiter plates (Costar^TM^, Corning, Tewksbury, MA, USA) were coated with 0.25 µg/well of protein extracts from each variety of mustard in carbonate-bicarbonate buffer (pH 9.6) and incubated overnight at 4 °C. Plates were blocked with 5% bovine serum albumin (BSA) in PBS-T for 2 h at room temperature followed by washing and incubation with control and mustard sensitive sera samples serially diluted in 1% BSA in PBS-T (dilution buffer) for another 2 h. For IgE detection, plates were washed and incubated 1 h in a 1:1000 (*v*/*v*) dilution of mouse anti-human IgE-HRP (clone B3102E8, Southern Biotech, AL, USA) prepared in dilution buffer. Bound peroxidase activity was determined with 3,3’,5,5’-tetramethylbenzidine (TMB) (Sigma-Aldrich, St Louis, MO, USA), the reaction was stopped by the addition of 1N sulfuric acid, and absorbance was measured at 450 nm. ELISA measurements were performed in duplicate.

### 2.6. Identification of Protein Bands as Allergens by LC-MS/MS

The protein in-gel digestion and the mass spectrometry experiments were performed by the Proteomics platform of the Eastern Quebec Genomics Center, Quebec, Canada. Detailed experimental parameters for the tryptic digestion, the mass spectrometry conditions, and the data analysis were previously reported by Rioux et al. [28]. Scaffold (Scaffold_3_00_07, Proteome Software Inc., Portland, OR, USA) was used to validate MS/MS-based peptide and protein identifications. Peptide identifications were accepted if they could be established at greater than 95.0% probability, as specified by the Peptide Prophet algorithm [29]. Protein identifications were accepted if they could be established at greater than 95.0% probability and contained at least two identified peptides. Protein probabilities were assigned by the Protein Prophet algorithm [30].

### 2.7. Protein Sequence Comparisons with Known and Putative Allergens

Each MS identified IgE-binding mustard protein sequence was compared to all proven and putative protein allergens sequences included in the Food Allergy Research and Resource Program (FARRP) AllergenOnline.org database version 19 (updated on 10 February 2019) [31]. This version contains a comprehensive list of 2129 protein (amino acid) sequence entries that are categorized into 853 taxonomic-protein groups of unique proven or putative allergens (food, airway, venom/salivary, and contact) from 384 species. All database entries are linked to sequences in the National Center for Biotechnology Information (NCBI) of the National Institute of Health (NIH). Sequence comparison was performed using the FASTA algorithm version 36 with a sliding window of 80 amino acid segments of each protein to find identities greater than 35%, as recommended by the CODEX Alimentarius guidelines [32]. The scoring matrix used on the AllergenOnline website is a BLOSUM 50 [33]. E-values and percent identities [(#identical residues/80 or more amino acids) * 100%)] were evaluated to consider potential cross-reactivity.

## 3. Results and Discussion

### 3.1. Protein Content and Extractability from Different Mustard Varieties

**Figure 1:**
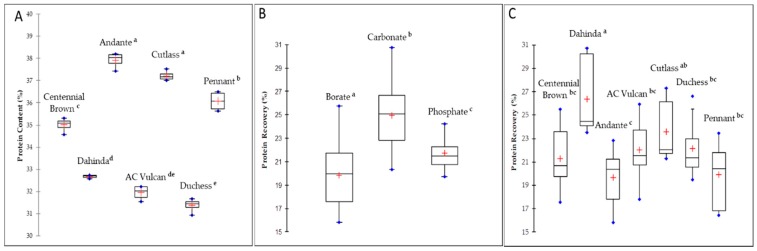
Protein content and extractability from different mustard varieties. Box plots represent the protein content of the studied Canadian mustard varieties (**A**), the effect of extraction buffer on mustard protein recovery (**B**), and the effect of mustard variety on protein recovery (**C**). Buffers or varieties with different superscripts are significantly different (Tukey multiple comparison of means, *p* < 0.0001).

The total seed protein content varied significantly (*p* < 0.0001) among the different mustard varieties, ranging from 36.07–37.92% for the two varieties of *Sinapis alba* (AC Pennant and Andante) and 31.38–37.22% for the five varieties of *Brassica juncea* (Duchess, AC Vulcan, Dahinda, Centennial Brown, and Cutlass), accounting for about 7% difference across varieties (Figure 1A). These values were generally higher than the mean protein values reported for Canadian mustard by the Canadian grain commission [11].

Regardless of the variety, carbonate buffer was significantly (*p* < 0.0001) more efficient than phosphate and borate buffers in solubilizing mustard proteins (Figure 1B). This result is in agreement with a previous study on *Brassicaceae* oilseeds that showed that solubility varied between species and varieties studied, while the highest value of N solubility was observed at pH 10 [34]. Similar results were also observed for peanut and tree-nut proteins, where carbonate was found to be the most efficient extracting buffer [35]. An alkaline medium allows more protein to be solubilized, and this effect seems to be the result of electrostatic repulsion. The most abundant protein fraction in crucifers is cruciferin [36], and since its isoelectric point (IP) is at 7.25 [37], this should explain why the electrostatic repulsion reaches a higher value in the carbonate buffer, thus giving better protein solubility.

Protein extractability also showed some significant variation according to mustard varieties. As presented in Figure 1C, *Brassica juncea* variety Dahinda exhibited the highest protein recovery (26.4%, *p* < 0.0001). Dahinda is a *Brassica juncea* canola quality variety, an oilseed that was developed with a low glucosinolate content but with oil equivalent to conventional canola species [38]. The breeding of this variety has also resulted in a high cruciferin content [39]. The reported low surface hydrophobicity of cruciferin at basic pH of 10 [40] might explain the higher solubility observed for this variety. On the opposite end, the *Sinapis alba* variety Andante showed the poorest extractability (19.7%, *p* < 0.0001), despite its high protein content (38%).

### 3.2. Protein Electrophoretic Profiles of Differents Mustard Varieties as a Function of Extraction Buffer

**Figure 2:**
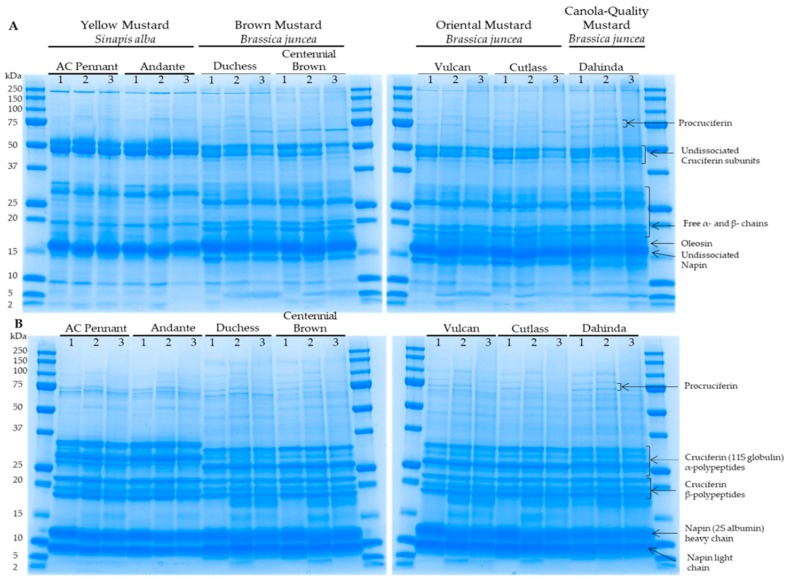
Protein electrophoretic profiles of mustard seed varieties as a function of extraction buffer. Proteins were separated under non-reducing (**A**) and reducing (**B**) conditions. 1: phosphate buffer; 2: borate buffer; 3: carbonate buffer. Molecular weights (MWs) of the standards are indicated in the left margin.

Polypeptide composition of the mustard varieties as a function of extraction buffer was resolved by gel electrophoresis in both non-reducing (Figure 2A) and reducing (Figure 2B) conditions. These figures revealed important qualitative differences in the protein profiles among the different mustard types/classes. In the presence of mercaptoethanol, the polypeptide profiles of the *Brassica* and the *Sinapis* varieties were similar to those obtained by Aluko et al. [41]. No major differences were found in the extracts’ electrophoretic profiles between the varieties of the same mustard type and for the same extraction buffer used (Figure 2A,B). A similar observation was made by Wanasundara et al. [34] for the solubility profile of cruciferin and napin between pH 2 and 10. However, differences in protein profiles were observed between the different buffer extracts for the same variety, suggesting that the buffer type affected mustard protein extraction not only quantitatively but also qualitatively. From the densitometry analysis of protein electrophoretic profile (Table 1), it was found that phosphate buffer tended to enhance napin protein extractability, particularly for the *B. juncea* varieties. Since napins have a high degree of polymorphism [36], it could be possible that some isoforms would show different solubility according to pH. In the case of *Brassica napus*, it was shown that napin was soluble at acidic, neutral, and basic pH, but that only a few isoforms were soluble at a pH of 8.5 [40]. In addition, the polypeptide profiles of borate and carbonate buffers extracts revealed the presence of protein bands at around 15 and 55 kDa, which were absent in the phosphate buffer extract. Moreover, an additional band of about 17 kDa, which was previously identified as an oleosin [42], was observed in borate and carbonate buffers extract under non-reducing conditions for the *B. juncea* varieties.

Based on these results, carbonate was identified as the most favorable extracting buffer because of its higher protein extraction capacity and more complete electrophoretic profile. Consequently, carbonate protein extracts were retained for the remainder of the study.

### 3.3. Indirect ELISA for Serum IgE Response to Mustard Varieties

**Figure 3:**
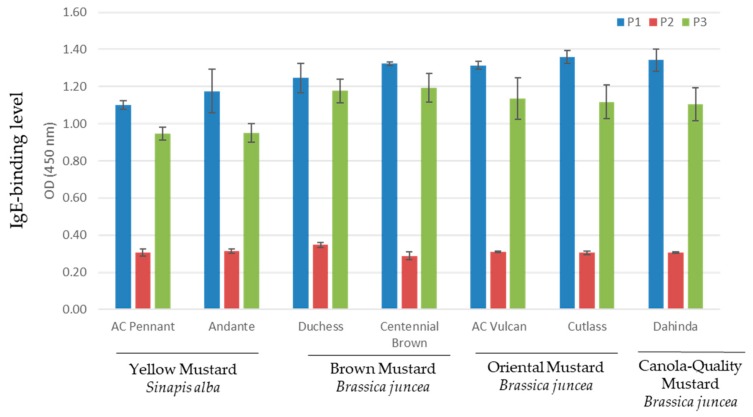
Indirect ELISA of specific Immunoglobulin E (IgE)-binding of different mustard protein to sera from mustard sensitive/allergic individuals.

Indirect ELISA was performed with individual serum (P1, P2, and P3) to compare the IgE-binding levels of the different *S. alba* and *B. juncea* varieties. As can be seen in Figure 3, all mustard varieties exhibited the highest binding to IgEs from serum P1, followed by serum P3. IgE-binding to serum P2 showed the lowest intensity. Differences in the intensity of mustard sensitive/allergic individuals are common and were reported in previous studies [5,7,8]. Although not statistically significant, the two sera P1 and P3 appeared to be less immunoreactive to *S. alba* varieties in comparison to the *B. juncea* varieties. Dust mite sensitized control sera did not bind to any mustard varieties used in this study (data not shown).

### 3.4. IgE-Binding Profiles of Select Canadian Mustard Varieties

**Figure 4:**
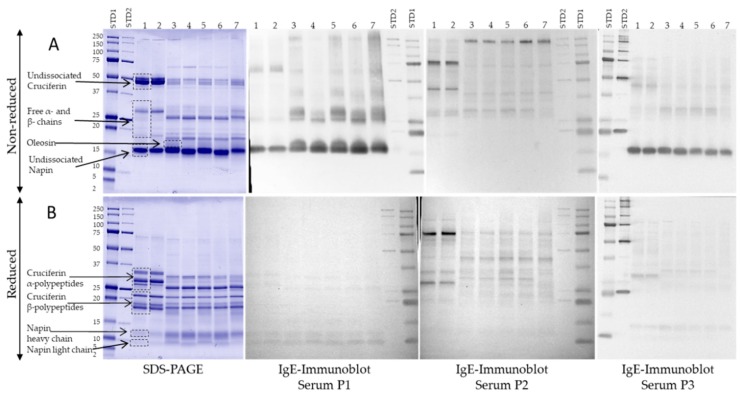
Protein and IgE- binding profiles of different mustard varieties. Carbonate mustard protein extracts were separated under non-reducing (**A**) and reducing (**B**) conditions. 1: *S. alba* AC Pennant; 2: *S. alba* Andante; 3: *B. juncea* Duchess; 4: *B. juncea* AC Vulcan; 5: *B. juncea* Dahinda; 6: *B. juncea* Centennial Brown; 7: *B. juncea* Cutlass; STD1 and STD2 are protein MW markers.

In order to evaluate the varietal effect on the IgE binding profiles of mustard proteins, immunoblotting with carbonate buffer extracts from the seven different mustard varieties was performed using sera from three mustard sensitive/allergic persons individually (Figure 4). IgE-immunoblotting was conducted for both non-reduced and reduced mustard proteins. The results revealed important differences in protein profile, abundance, and IgE-binding intensity between the *S. alba* and the *B. juncea* mustard types as well as in the IgE-reactivity profiles between the three sera. According to Menendez-Arias et al. [13], Sin a 1 (2S albumin) is the major allergen of *S. alba* seeds and Bra j1 of *B. juncea* seeds [23]. Under non-reduced conditions (Figure 4B), the 2S albumins showed intense IgE-binding in the case of sera P1 and P3. However, under reduced electrophoretic conditions (Figure 4A), these two sera only weakly bound the two napins bands. This observation suggests the recognition of conformational epitopes. A previous study [14] identified two epitopes of Sin a 1—one conformational and one linear. According to Monsalve et al. [24], it is the linear epitope that is considered to be the antigenic determinant. Based on this epitope, an anti-epitope antibody for the quantification of Sin a 1 by a non-competitive enzyme linked immunosorbent assay was developed [43]. This same study also showed that the napin protein fraction of yellow mustard contained proteins devoid of the linear epitope sequence, thereby not contributing to all cases of 2S allergenicity. In the case of serum P2, there was no evidence of IgE-binding to napin proteins under both reduced and non-reduced conditions. A previous study [44] also showed that, even though the majority of mustard allergic persons reacted to Sin a 1, some patients’ sera did not bind to the 2S albumin. This observation could be explained by the finding that Sin a 1 presents an important polymorphism [45], resulting in significant variability in its allergenic potential.

IgE-binding pattern of the mustard sensitized sera revealed the presence of other reactive protein bands from both *S. alba* and *B. juncea* varieties. For the latter, sera P1 and P3 exhibited IgE-binding on the non-reduced immunoblots for polypeptide bands between 27 and 48 kDa and on reduced ones for bands between 22 and 34 kDa. These regions corresponded to cruciferin (11S globulin) and, in the case of *S. alba* seeds, to the allergen Sin a 2 [19]. To date, no reported allergen has been recorded for the 11S of *B. juncea* seeds. However, a bioinformatics evaluation of the cruciferin of *B. juncea*, *B. napus*, and *S. alba* showed that the cruciferin of *B. juncea* has a high similarity to the one of *S. alba* [46]. As a result of this high homology, it would be possible for the 11S globulin of *B. juncea* to present an allergic potential, but this still has to be clinically demonstrated. The IgE-binding to the 11S globulin was less intense than for the 2S albumin in the case of sera P2 and P3. Similar results were obtained by Menendez-Arias et al. [13]. In addition, for serum P1, intense IgE binding was observed in *B. juncea* varieties to bands between 27 and 31 kDa on the non-reduced immunoblot, also corresponding to the free polypeptide chains of the cruciferin. In the case of the two *S. alba* varieties, binding was observed at a polypeptide band around 60 kDa. However, in both cases, the binding to cruciferin was not observed on the reduced immunoblot. This is contrary to what was previously published about the 11S cruciferin of *S. alba* reporting that, under reducing conditions, the two subunits of the protein were able to bind IgE from the sera [19]. Such a difference is probably due to the variability in the sensitization profile of the used sera; moreover, that study involved the use of pooled sera, and a different result could have been obtained if the sera were used individually. A strong IgE-binding was further observed on both the reduced and the non-reduced immunoblots of serum P2 for a procruciferin band with molecular weight of about 75 kDa. It was reported [36] that it is common to observe polypeptide bands that remain at apparent molecular weight above 54 kDa, presumably from the precursor polypeptide or procruciferins of α-β, which have not undergone regular in vivo processing [47,48]. Finally, sera P1 bound strongly to the *B. juncea* protein around 17 kDa, while sera P2 and P3 showed binding to a band around 55 kDa on the reduced immunoblot for the *S. alba* varieties. This last band was not observed on the electrophoretic profile for the phosphate buffer extracts. However, it appeared on the profiles for the borate and the carbonate extracts, thus confirming the importance of choosing a buffer that provides the most complete protein profile so as to increase the probability of revealing as many IgE reactive bands as possible. All IgE-reactive proteins were further subjected to LC-MS analysis for identification.

### 3.5. Identification of Mustard IgE-Binding Proteins by Mass Spectroscopy

**Figure 5:**
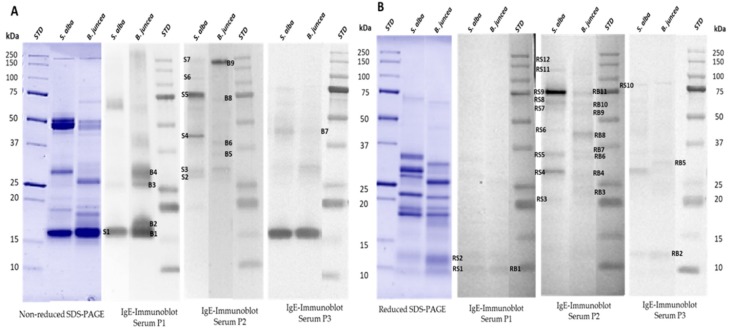
Identification of mustard IgE-binding protein bands for in gel-tryptic digestion and LC-MS/MS analysis. Protein extracts from *S. alba* AC Pennant and *B. juncea* AC Vulcan were separated under non-reducing (**A**) and reducing (**B**) conditions and immunoblotted using sera P1, P2, and P3. STDs are protein MW markers.

To confirm the identity of the IgE-binding bands, electrophoretic analyses of mustard varieties AC Pennant (*S. alba*) and AC Vulcan (*B. juncea*) were run again, and the immunoreactive bands were excised and further analyzed by LC/ESI-MS/MS. Figure 5 represents the SDS-PAGE and the immunoblot patterns of the two mustard varieties incubated with sera P1, P2, and P3. The list of MS identified proteins is presented in Table 2. All the allergenic protein bands (as shown in Figure 5A,B) were identified by MS/MS analysis as belonging to the *Brassicaceae* family. Excised bands S1, RS1, and RS2 of *S. alba* and bands B1, RB1, and RB2 from *B. juncea* from both non-reduced and reduced gels were identified as Sin a 1 (*S. alba*) and Braj 1 (*B. juncea*), respectively, thereby confirming their allergen identity. As for B2 and RB4 bands, these were identified as oleosin proteins (OLES2_BRANA Oleosin S2-2 and BRANA Oleosin S3-1) from the database (UniProtKB/Swiss-Prot and UniProtKB/TrEMBL; www.uniprot.org). Although this is the first formal evidence of the allergenicity of such oil bodies-associated proteins as potential allergens from mustard, future studies need to be performed to prove the biological activity of these newly identified allergens. Recent work has shown that two oil body-associated proteins [Oleosins, Ses i 4 (17 kDa) and Ses i 5 (15 kDa)] were found to be among the most important sesame allergens [49]. Allergenic oleosins were also reported in peanuts, where five different IgE-binding oleosins with a molecular weight from 14–18 kDa were identified [50,51,52], while two oleosin isoforms of 17 and 14–16 kDa, now designated Cor a12 and Cor a13, were identified as allergens in hazelnut [53]. Oleosin proteins were also identified from B1, B2, B3, and B4, suggesting the presence of multiple isoforms of the proteins.

Subunit bands assigned as S4 to S7 and RS3–RS12 from AC Pennant (*S. alba*) in addition to B3–B8 and RB3, RB5–RB9, and RB11 from AC Vulcan (*B. juncea*) mustard varieties were identified and confirmed as cruciferin (11S globulin) fragments. The presence of several cruciferin (α-β) polypeptides that form the subunits (protomers) of the 11S globulin molecule in mustard has been reported [36]. However, not all of them have been characterized yet as potential mustard allergens within the 11S globulin family. To date, the only 11S globulin storage protein that has been identified as an important mustard allergen is Sin a 2 [19]. Future studies need to be performed to characterize these newly identified allergens and name them in accordance with the World Health Organization/International Union of Immunological Societies (WHO/IUIS) Allergen Nomenclature Subcommittee [54].

Furthermore, the protein bands S7 and RS9 from *S. alba* as well as B9 and RB10 from *B. juncea* were identified as β-glucosidase precursors (BRANA, accession No. Q42618) showing 44–50% sequence coverage. Indeed, a β-glucosidase was previously purified from seeds of *B. napus* (oilseed rape) as reported by Falk and Rask [55]. The 130 kDa native enzyme consisted of a disulfide linked dimer of 64 kDa monomers. Evidence was previously reported about the potential allergenicity of a β-glucosidase from wheat [56]. The protein band RB9 was also identified with 64% coverage and 21 unique peptides as an enolase (BRACM, accession No. Q6W7E8). Enolase is an essential glycolytic enzyme that catalyzes the interconversion of 2-phosphoglycerate and phosphoenolpyruvate [57]. It has been recognized as an important allergen from various molds and some plants [58,59,60]. Finally, in addition to oleosin, the RB4 band was also identified as a glutathione-S transferase (GST) (accession No. Q7XZT2). Members of the GST family have been reported as relevant allergens in cockroach [61], fungi [62], and wheat [63]. The allergenicity of these new identified IgE-binding proteins from mustard would request further investigation and should be carefully evaluated not only by in vitro IgE tests but also by in vivo and clinical tests.

### 3.6. Bioinformatic Assessment of Potential Cross-Reactivity of Identified Mustard IgE-Binding Proteins with Known Allergens

The purpose of this analysis was to identify relevant homology in amino acid sequences between the identified mustard IgE-binding proteins and proven or putative allergens, which could help identify proteins that may share immunologic or allergic cross-reactivity. The Food Allergy Research and Resource Program (FARRP) AllergenOnline.org database version 19 (updated on 10 February 2019; http://www.allergenonline.org/) was used for the primary comparisons to allergens. This public database only shows sequences of proteins with sufficient published evidence of allergy at a minimum-specific IgE binding from sera of subjects allergic to the source [31]. Based on the recommendation of the CODEX Alimentarius guidelines [32], the FASTA3 algorithm with the criteria of >35% identity over any segment of 80 or more amino acids as an indication of possible cross-reactivity for allergens was used to compare all possible contiguous amino acid segments of each of the identified mustard IgE-binding proteins against all sequences listed in the AllergenOnline Database. Research has, however, shown that proteins with greater than 70% identical primary amino acid sequences throughout the length of the protein are commonly cross-reactive, while those with less than 50% identity are unlikely to be cross-reactive [64]. For increased confidence, only the best scoring matches (>35% identity) with E-values smaller than 1e-7 are displayed in Table 3, as it has been reported that larger E-values are unlikely to identify relevant matches, while matches with E-values smaller than 1e-30 are much more likely to be cross-reactive in at least some allergic individuals [65]. The complete search results are presented in a supplemental document (Appendix 1). The 80mer FASTA search confirmed the extensive homology and the high cross-reactivity between the 2S albumins from *Sinapis alba* (Sin a 1) and *Brassica* species (Bra j 1) with a percentage of identity (ID) over 87% and very small E-value. Interestingly, FASTA identified highly significant alignments (>55% identity) of the IgE-binding 11S globulins (cruciferins) from both *Sinapis* and *Brassica* species (accession No. Q2TLWO, Q7XB53, P33525, Q2TLV9) with 11S globulins allergens from tree-nut species, most notably with black walnut (*Juglans nigra*), cashew (*Anacardium occidentale*), hazelnut (*Corylus avellana*), almond (*Prunus dulcis*), and pecan (*Carya illinoinensis*), suggesting a strong possibility of cross-reactivity, which would be worth testing using sera from individuals with clinical reactivity to those species.

In addition, the search identified probable homology for 11S globulin proteins from *Sinapis alba* (accession No. Q2TLWO and Q2TLV9) with high molecular weight (HMW) glutenin from wheat (*Triticum aestivum*) based on 60% and 68% best identity and low E-values of 1.3e-12 and 3.5e-18, respectively. Noteworthy, sequence alignments for the newly identified IgE-binding mustard oleosins (accession No. C3S7F1 and C3S7F8) found highly significant matches with oleosin allergens from hazelnut (*Corylus avellana*) with ID over 70% and E-values smaller than 1e-30. These results are highly relevant for potential cross-reactivity. Significant scores were also found with oleosins from peanut (*Arachis hypogaea*) and sesame (*Sesamum indicum*). Scoring results for the identified enolase protein from *Brassica juncea* (accession No. Q6W7E8) resulted in the best alignments (over 80% ID and very small E-value) to enolase allergens from latex tree (*Hevea brasiliensis*), yellowfin tuna (*Thunnus albacares*), Atlantic salmon (*Salmo salar*), chicken (*Gallus gallus*), and fungi (*Candida albicans* and *Rhodotorula mucilaginosa*). This finding adds to the existing knowledge that enolase is one of the most conserved glycolytic enzyme protein across eukaryotes (animal, plant, and fungi) [58,59]. This further suggests a strong possibility of cross-reactivity. Besides, the identified mustard Glutathione S-transferase 3 (GST) enzyme (accession No. Q7XZT2) also significantly matched GST allergen Per a 5 from the insect American cockroach (*Periplaneta americana*), with 40% ID and an E-value of 1.2e-6. Finally, other identified mustard proteins with accession No. Q42618 (β-glucosidase), O23733 (Cysteine synthase), P13244 (malate synthase, glyoxysomal), and C3S7H5 (caleosin) resulted in no matches greater than 35% identity over 80 amino acids.

## 4. Conclusions

In this study, carbonate buffer was found as an efficient non-denaturing buffer for the extraction of mustard seed proteins. Protein and IgE-binding patterns revealed important differences between *S. alba* and *B. juncea* types of mustard, but no major differences were observed between the varieties of the same mustard type. The presence of both napins (2S albumins) and cruciferins (11S globulins) allergenic polypeptides under both non-reducing and reducing electrophoretic conditions was confirmed. Sin a 1, Bra j 1, and cruciferin polypeptides exhibited a stronger IgE reactivity under non-reducing conditions in comparison to reducing conditions, demonstrating the presence of conformational allergenic epitopes in Sin a 1, Bra j 1, and other cruciferin subunits. Therefore, the use of denaturing protein extraction and analysis conditions may lead to failure to detect important immuno-reactive epitopes due to protein modification. Results also revealed the presence, in both *S. alba* and *B. juncea* types of mustard, of a wide range of IgE binding cruciferin (11S globulin) polypeptides/fragments with different molecular weight, indicating the existence of multiple isoforms in all types of mustard seeds. We also reported, for the first time, new mustard IgE binding polypeptides/proteins identified as oil body-associated proteins (oleosins) and enolase enzyme from *B. juncea* type. A bioinformatics analysis to identify relevant homology in amino acid sequences between the identified mustard IgE-binding proteins and known allergens revealed strong possible cross-reactivity between mustard 11S globulin and equivalent allergen proteins from tree-nut species and wheat. Strong cross-reactivity between mustard oleosins with those of hazelnut, peanut, and sesame was also suggested. In addition, a highly significant homology between mustard enolase and that of other eukaryotes (animal, plant, and fungi) was found, confirming the highly conserved structure of this glycolytic enzyme and its high potential for allergic cross-reactions. The new putative mustard allergens revealed in this study would request further biological and structural characterization.

## Figures and Tables

**Table 1:** Densitometry analysis of electrophoretic profiles of mustard protein extracts.

Band Intensity (1 × 10^6^)																								
		*Sinapis alba*								*Brassica juncea*														
	MW ^a^	AC Pennant			Andante				MW ^a^	Duchess			Centennial Brown			Vulcan			Cutlass			Dahinda		
Band Name	kDa	1	2	3	1	2	3	Band Name	kDa	1	2	3	1	2	3	1	2	3	1	2	3	1	2	3
Sin a 1 LC ^b^	9.5	24.1	18.9	19.1	23.4	18.3	18.3	Bra j 1 LC ^b^	9.5	23.7	15.0	19.6	23.7	13.0	15.7	22.4	18.1	19.9	27.2	16.7	21.2	26.4	23.1	22.5
Sin a 1 HC ^c^	12	25.0	19.2	19.4	11.8	19.4	19.1	Bra j 1 HC ^c^	12	19.2	11.1	14.1	17.7	10.0	18.0	13.9	12.9	14.7	19.6	10.8	15.0	16.1	13.9	14.6
Unknown	15.1		0.6	2.8		0.8	2.1	Unknown	14.6		2.4	1.2		3.3	1.1		2.8	1.1		3.7	0.8			
Cruciferin								Cruciferin	16.8		7.1	6.8		5.5	5.4		7.3	8.2		6.8	6.0	3.5	10.5	9.9
β-polypeptides	18	13.3	14.8	15.0	11.8	14.2	10.6	β-polypeptides	18	11.3	10.3	8.7	12.7	11.8	10.9	13.9	12.3	9.7	14.8	12.5	11.8	13.7	14.3	13.3
11 S globulin	19.1	5.9	11.6	9.6	6.4	12.0	5.1	11 S globulin	19.1	5.7	6.7	5.2	5.7	6.4	5.3	6.6	7.9	5.9	5.7	6.5	4.7	6.7	8.2	7.0
Sin a 2	21.6	8.8	12.6	10.5	9.0	12.5	7.8	Sin a 2	20.9	6.3	6.1	5.2	7.5	7.1	6.5	7.2	6.5	5.2	7.2	6.4	5.4	7.9	7.7	6.7
Cruciferin								Cruciferin	23.7	1.4	1.3		2.3	2.2	1.7	2.2	1.9	1.6	2.2	1.9	1.6	2.6	2.7	2.7
α-polypeptides	26.8	6.2	7.0	6.5	4.4	4.2	4.7	α-polypeptides	25.3	10.5	10.4	10.4	13.0	12.6	12.1	13.7	12.5	10.7	14.1	12.9	11.9	11.1	10.4	10.0
11S globulin	28.4	10.6	13.2	11.7	11.7	14.1	12.3	11S globulin	28	2.6	2.3	1.6	2.8	3.1	2.5	2.7	3.0	2.1	2.8	2.8	2.1	3.7	3.5	3.1
Sin a 2	30.9	1.3			0.8			Sin a 2	30.5	9.6	9.1	7.3	12.0	10.6	9.8	10.2	8.7	7.2	10.2	8.7	7.2	6.2	5.6	5.0
	32.9	10.5	11.0	9.4	10.0	10.4	9.3																	
11S fragment	55		1.7	1.5		1.9	1.6	11S fragment	52		1.0	0.5	1.1	1.4	1.3	0.8	1.0	0.9	1.1	1.2	1.0	1.8	2.4	1.3
Procruciferin	63.5	1.0	1.0	1.1	0.9	1.6	3.0	Procruciferin	63.6	2.9			1.1	1.6	1.5	1.3	1.5	1.7	1.7	1.3	1.2	2.0	3.0	1.0
	66.8	2.0			1.5	2.0	2.7		65.9		3.8	2.6				2.3		1.5	1.1	1.1	1.3	1.2		
	69.4	3.4	5.9	5.2	2.0	3.2	2.0		69.9	2.0			1.8	1.2	1.1		1.9					2.4	2.0	1.2
	73.9	2.7	2.5	1.7	2.3	3.3			72.5		2.7	1.0		1.3		2.8	2.3		2.4	1.4			3.4	1.2

^a^ Based on reduced SDS-PAGE; ^b^ light chain; ^c^ heavy chain; 1: phosphate buffer; 2: borate buffer; 3: carbonate buffer.

**Table 2:** Identification of IgE-binding proteins from *Sinapis alba* and *Brassicae juncea* mustard by mass spectrometry analysis.

Band Number ^a^	MW on SDS-PAGE (KDa)	Protein Name ^b^	Protein Accession Numbers	Protein MW (KDa)	Exclusive Unique Peptide Count	Percentage Sequence Coverage ^c^
***Sinapis alba* (AC Pennant)**						
S1	16	ALL1_SINAL Allergen Sin a 1 [Sinapis alba (White mustard)]	P15322	16.4	5	23.40%
S2	28	SINAL 11S globulin [Sinapis alba (White mustard)]	Q2TLW0	56.5	16	39.40%
S3	30	SINAL 11S globulin [Sinapis alba (White mustard)]	Q2TLW0	56.5	16	39.40%
		CRU4_BRANA Cruciferin CRU4 [Brassica napus (Rape)]	P33522	51.4	13	30.10%
S4	44	SINAL 11S globulin [Sinapis alba (White mustard)]	Q2TLW0	56.5	19	44.30%
		CRU4_BRANA Cruciferin CRU4 [Brassica napus (Rape)]	P33522	51.4	14	29.70%
S5	75	SINAL 11S globulin [Sinapis alba (White mustard)]	Q2TLW0	56.5	18	44.30%
		CRU4_BRANA Cruciferin CRU4 [Brassica napus (Rape)]	P33522	51.4	7	23.40%
S6	107	SINAL 11S globulin [Sinapis alba (White mustard)]	Q2TLW0	56.5	21	46.50%
S7	223	SINAL 11S globulin [Sinapis alba (White mustard)]	Q2TLW0	56.5	20	39.40%
		BRANA Beta-glucosidase [Brassica napus (Rape)]	Q42618	56.5	20	34.40%
RS1	9.5	ALL1_SINAL Allergen Sin a 1 [Sinapis alba (White mustard)]	P15322	16.4	7	26.90%
RS2	12	ALL1_SINAL Allergen Sin a 1 [Sinapis alba (White mustard)]	P15322	16.4	9	57.90%
RS3	20	SINAL 11S globulin [Sinapis alba (White mustard)]	Q2TLW0	56.5	19	36.10%
		BRANA Cruciferin (Fragment) [Brassica napus (Rape)]	Q7XB53	51.3	8	23.20%
RS4	28	SINAL 11S globulin [Sinapis alba (White mustard)]	Q2TLW0	56.5	14	25.90%
		CRU3_BRANA Cruciferin CRU1 [Brassica napus (Rape)]	P33525	56.5	7	27.50%
RS5	34	SINAL 11S globulin [Sinapis alba (White mustard)]	Q2TLW0	56.5	14	25.90%
		SINAL 11S globulin [Sinapis alba (White mustard)]	Q2TLV9	57.9	5	34.60%
RS6	44	SINAL 11S globulin [Sinapis alba (White mustard)]	Q2TLW0	56.5	18	46.70%
RS7	55	SINAL 11S globulin [Sinapis alba (White mustard)]	Q2TLW0	56.5	15	37.60%
		CRU4_BRANA Cruciferin CRU4 [Brassica napus (Rape)]	P33522	51.4	10	25.20%
RS8	62	SINAL 11S globulin [Sinapis alba (White mustard)]	Q2TLW0	56.5	20	45.10%
RS9	75	Q2TLW0_SINAL 11S globulin [Sinapis alba (White mustard)]	Q2TLW0	56.5	16	40.20%
		BRANA Beta-glucosidase [Brassica napus (Rape)]	Q42618	58.5	12	20.80%
RS10	82	Q2TLW0_SINAL 11S globulin [Sinapis alba (White mustard)]	Q2TLW0	56.5	14	37.10%
RS11	118	SINAL 11S globulin [Sinapis alba (White mustard)]	Q2TLW0	56.5	12	27.50%
RS12	140	SINAL 11S globulin [Sinapis alba (White mustard)]	Q2TLW0	56.5	9	24.50%
***Brassicae juncea* (AC Vulcan)**						
B1	15.5	Allergen Bra j 1-E [Brassica juncea (Indian mustard)]	P80207	14.6	5	51.90%
		OLES2_BRANA Oleosin S2-2 [Brassica napus (Rape)]	C3S7F1	19.9	15	46.80%
B2	17	BRANA Oleosin S3-1 [Brassica napus (Rape)]	C3S7F8	19.6	10	41.70%
		OLES2_BRANA Oleosin S2-2 [Brassica napus (Rape)]	C3S7F1	19.9	16	46.80%
B3	26	BRANA Caleosin CLO1-2 [Brassica napus (Rape)]	C3S7H5	28.1	7	35.90%
		CRU4_BRANA Cruciferin CRU4 [Brassica napus (Rape)]	P33522	51.4	21	49.50%
B4	30	CRU3_BRANA Cruciferin CRU1 [Brassica napus (Rape)]	P33525	56.5	12	47.20%
		OLES2_BRANA Oleosin S2-2 [Brassica napus (Rape)]	C3S7F1	19.9	11	46.30%
B5	29	BRANA Cruciferin (Fragment) [Brassica napus (Rape)]	Q7XB53	51.3	4	8.80%
		CRU4_BRANA Cruciferin CRU4 [Brassica napus (Rape)]	P33522	51.4	8	24.30%
B6	34	SINAL 11S globulin [Sinapis alba (White mustard)]	Q2TLW0	56.5	7	20.80%
		CRU3_BRANA Cruciferin CRU1 [Brassica napus (Rape)]	P33525	56.5	3	21.80%
B7	42.5	CRU3_BRANA Cruciferin CRU1 [Brassica napus (Rape)]	P33525	56.5	12	45.20%
		CRU4_BRANA Cruciferin CRU4 [Brassica napus (Rape)]	P33522	51.4	20	52.30%
B8	68	CRU3_BRANA Cruciferin CRU1 [Brassica napus]	P33525	56.5	14	52.80%
		CRU4_BRANA Cruciferin CRU4 [Brassica napus]	P33522	51.4	13	40.90%
B9	200	Malate synthase. glyoxysomal [Brassica napus (Rape)]	P13244	63.7	14	23.00%
		BRANA Beta-glucosidase [Brassica napus (Rape)]	Q42618	58.5	23	44.20%
RB1	9.5	Allergen Bra j 1-E [Brassica juncea (Indian mustard)]	P80207	14.6	2	38.00%
RB2	12	Allergen Bra j 1-E [Brassica juncea (Indian mustard)]	P80207	14.6	5	68.20%
		ALL1_SINAL Allergen Sin a 1 [Sinapis alba (White mustard)]	P15322	16.5	4	35.20%
RB3	22	CRU3_BRANA Cruciferin CRU1 [Brassica napus (Rape)]	P33525	56.5	11	39.50%
		CRU4_BRANA Cruciferin CRU4 [Brassica napus (Rape)]	P33522	51.4	11	35.30%
RB4	27	BRANA Caleosin CLO1-2 [Brassica napus (Rape)]	C3S7H5	28.1	8	37.60%
		OLES2_BRANA Oleosin S2-2 [Brassica napus (Rape)]	C3S7F1	20.0	6	40.40%
		BRAJU Glutathione S-transferase 3 [Brassica juncea]	Q7XZT2	24.1	7	46.00%
RB5	30	CRU4_BRANA Cruciferin CRU4 [Brassica napus (Rape)]	P33522	51.4	17	42.60%
RB6	30	CRU3_BRANA Cruciferin CRU1 [Brassica napus (Rape)]	P33525	56.5	12	45.20%
		CRU4_BRANA Cruciferin CRU4 [Brassica napus (Rape)]	P33522	51.4	15	38.70%
RB7	34	CRU3_BRANA Cruciferin CRU1 [Brassica napus (Rape)]	P33525	56.5	8	28.90%
		BRAJU Cysteine synthase [Brassica juncea]	O23733	33.9	8	33.20%
RB8	47	CRU4_BRANA Cruciferin CRU4 [Brassica napus (Rape)]	P33522	51.4	25	54.00%
		BRANA Cruciferin (Fragment) [Brassica napus (Rape)]	Q7XB53	51.3	14	39.70%
RB9	52	CRU4_BRANA Cruciferin CRU4 [Brassica napus (Rape)]	P33522	51.4	16	45.40%
		BRACM Enolase [Brassica campestris (Field mustard)]	Q6W7E8	47.4	21	61.00%
RB10	62	BRANA Beta-glucosidase [Brassica napus (Rape)]	Q42618	58.5	13	30.00%
RB11	76	CRU4_BRANA Cruciferin CRU4 [Brassica napus (Rape)]	P33522	51.4	8	30.10%
		SINAL 11S globulin [Sinapis alba (White mustard)]	Q2TLW0	56.5	5	15.30%

**^a^** Band numbers correspond to the IgE-binding bands detected in Figure 5; **^b^** only protein identifications with 100% probability were retained; **^c^** total percentage of proteins amino acid sequence covered by the identified peptides in MS/MS analysis.

**Table 3:** Significant sequence alignment of the identified mustard IgE-binding proteins with allergens from the Allergen Online database.

Protein Identification ^a^	Species	Best %ID ^b^	# Hits >35%	Full Alignment			NCBI Links ^f^
				E-value ^c^	%ID ^d^	Length ^e^	
**P15322: Allergen Sin a 1 [Sinapis alba (White mustard)]**							
gid|192|Allergen Allergen Sin a 1 precursor	*Sinapis alba*	100.00%	66 of 66	2.4e-34	100.0%	145	gi|51338758
gid|1172|Putative 2S storage protein	*Brassica rapa*	93.80%	66 of 66	2.5e-29	88.30%	145	gi|17697
gid|1142|Putative Napin-3	*Brassica napus*	91.20%	66 of 66	2.6e-17	82.60%	144	gi|75107016
gid|1170|Putative Allergen Bra j 1-E	*Brassica juncea*	87.50%	66 of 66	1.8e-17	79.90%	144	gi|32363444
**P80207: Allergen Bra j 1-E [Brassica juncea (Indian mustard)]**							
gid|1170|Putative Allergen Bra j 1-E (Bra j 1)	*Brassica juncea*	100.00%	50 of 50	7.1e-23	100.0%	129	gi|32363444
gid|1142|Putative Napin-3 (Napin BnIII)	*Brassica napus*	92.50%	50 of 50	8.9e-20	89.10%	129	gi|75107016
gid|192|Allergen allergen sin a 1.0104	*Sinapis alba*	88.78%	50 of 50	2.5e-13	80.60%	144	gi|1009434
gid|1172|Putative 2S storage protein	*Brassica rapa*	85.00%	50 of 50	1e-11	77.10%	144	gi|17697
gid|386|Putative recombinant Ib pronapin	*Brassica napus*	55.00%	50 of 50	3.8e-6	50.00%	114	gi|26985163
**Q2TLWO: 11S globulin [Sinapis alba (White mustard)]**							
gid|837|Allergen 11S globulin precursor	*Sinapis alba*	100.00%	431 of 431	0	100.0%	510	gi|62240390
gid|2066|Putative 11S legumin protein	*Carya illinoinensis*	62.51%	351 of 431	2.6e-61	43.20%	521	gi|158998782
gid|2597|Putative legumin	*Juglans nigra*	62.51%	362 of 431	2.7e-58	42.00%	528	gi|1126299828
gid|76|Allergen allergen Ana 0 2	*Anacardium occidentale*	60.04%	380 of 431	2.3e-70	44.80%	498	gi|25991543
gid|160|Allergen HMW glutenin	*Triticum aestivum*	60.00%	84 of 431	3.5e-18	37.60%	213	gi|288860106
gid|392|Allergen 11S globulin	*Corylus avellana*	58.00%	387 of 431	3.6e-61	45.70%	534	gi|18479082
gid|1572|Putative prunin 2 precursor	*Prunus dulcis*	57.80%	418 of 431	3e-61	45.60%	522	gi|307159114
gid|2650|Allergen 11S globulin-	*Actinidia chinensis*	57.50%	352 of 431	2.3e-68	41.40%	503	gi|82469930
gid|2274|Putative 11S globulin	*Sesamum indicum*	55.00%	342 of 431	3.7e-60	42.10%	470	gi|13183173
gid|1093|Putative 11S globulin	*Pistacia vera*	55.00%	356 of 431	6.5e-53	41.80%	505	gi|156001070
gid|531|Putative allergenic protein	*Fagopyrum tataricum*	55.00%	256 of 431	1.5e-43	38.10%	486	gi|113200131
gid|733|Putative glycinin subunit G3	*Glycine max*	53.10%	272 of 431	2e-38	36.20%	514	gi|18639
gid|347|Putative 11S globulin	*Bertholletia excelsa*	52.50%	318 of 431	5.8e-59	39.20%	510	gi|30313867
**Q7XB53: Cruciferin (Fragment) [Brassica napus (Rape)]**							
gid|837|Allergen 11S globulin precursor	*Sinapis alba*	76.50%	387 of 387	3.5e-29	61.00%	472	gi|62240390
gid|392|Allergen Cor a 9 allergen	*Corylus avellana*	63.70%	353 of 387	9.4e-22	44.00%	509	gi|557792009
gid|1572|Putative Pru du 6 allergen	*Prunus dulcis*	62.51%	160 of 387	3.2e-10	48.20%	193	gi|523916668
gid|76|Allergen allergen Ana 0 2	*Anacardium occidentale*	61.30%	333 of 387	1.2e-22	44.70%	474	gi|25991543
gid|2597|Putative legumin	*Juglans nigra*	58.79%	298 of 387	3.8e-21	42.70%	503	gi|1126299828
gid|2066|Putative 11S legumin protein	*Carya illinoinensis*	58.79%	326 of 387	3.1e-21	43.50%	506	gi|158998782
gid|817|Putative seed storage protein	*Juglans regia*	57.52%	325 of 387	4.4e-21	42.90%	513	gi|56788031
gid|1093|Putative Pis v 2.0201 allergen 11S	*Pistacia vera*	56.30%	347 of 387	1.7e-33	41.60%	459	gi|110349085
gid|347|Putative 11S globulin	*Bertholletia excelsa*	53.80%	349 of 387	1.2e-24	43.30%	466	gi|30313867
gid|733|Putative glycinin subunit G3	*Glycine max*	53.80%	274 of 387	4.8e-22	38.00%	479	gi|18639
gid|2274|Putative 11S globulin	*Sesamum indicum*	52.46%	318 of 387	1.6e-24	40.80%	476	gi|13183173
gid|291|Allergen trypsin inhibitor	*Arachis hypogaea*	49.40%	59 of 387	1.9e-5	35.80%	204	gi|22135348
**P33525: Cruciferin CRU4 [Brassica napus (Rape)**							
gid|837|Allergen 11S globulin precursor	*Sinapis alba*	98.80%	430 of 430	2.4e-210	91.60%	510	gi|62240390
gid|2066|Putative 11S legumin protein	*Carya illinoinensis*	65.00%	358 of 430	1.9e-61	44.70%	535	gi|158998782
gid|2597|Putative legumin	*Juglans nigra*	63.79%	373 of 430	4.1e-61	43.30%	533	gi|1126299828
gid|817|Putative seed storage protein	*Juglans regia*	63.79%	368 of 430	1e-63	44.50%	533	gi|56788031
gid|1572|Putative Pru du 6 allergen	*Prunus dulcis*	60.04%	168 of 430	4.6e-20	49.70%	195	gi|523916668
gid|76|Allergen allergen Ana 0 2	*Anacardium occidentale*	60.04%	379 of 430	7.9e-73	46.20%	487	gi|25991543
gid|392|Allergen Cor a 9 allergen	*Corylus avellana*	58.79%	391 of 430	4.6e-62	45.50%	528	gi|557792009
gid|160|Allergen glutenin	*Triticum aestivum*	56.26%	70 of 430	2.5e-11	33.30%	207	gi|736319
gid|1093|Putative Pis v 2.0101 11S globulin	*Pistacia vera*	55.00%	345 of 430	6.7e-51	41.10%	518	gi|110349083
gid|2274|Putative 11S globulin	*Sesamum indicum*	53.80%	343 of 430	4.8e-63	42.10%	478	gi|13183173
gid|531|Putative allergenic protein	*Fagopyrum tataricum*	53.80%	255 of 430	4.4e-44	35.60%	523	gi|113200131
gid|347|Putative 11S globulin	*Bertholletia excelsa*	52.50%	309 of 430	2.3e-59	39.70%	506	gi|30313867
gid|733|Putative glycinin subunit G3	*Glycine max*	51.20%	267 of 430	3e-39	36.00%	516	gi|18639
gid|291|Allergen allergen Arah3/Arah4	*Arachis hypogaea*	47.50%	219 of 430	2.1e-25	33.20%	548	gi|21314465
gid|574|Putative glycinin precursor	*Glycine max*	47.50%	116of430	4.9e-28	39.80%	191	gi|169971
**Q2TLV9: 11S globulin [Sinapis alba (White mustard)]**							
gid|837|Allergen 11S globulin precursor	*Sinapis alba*	100.00%	444 of 444	0	100.00%	523	gi|62240392
gid|160|Allergen HMW glutenin	*Triticum aestivum*	68.80%	99 of 444	1.3e-12	38.60%	228	gi|288860106
gid|2066|Putative 11S legumin protein	*Carya illinoinensis*	63.79%	360 of 444	6.3e-41	41.90%	532	gi|158998782
gid|817|Putative seed storage protein	*Juglans regia*	62.51%	367 of 444	1.1e-41	41.70%	545	gi|56788031
gid|2597|Putative legumin	*Juglans nigra*	62.51%	364 of 444	2.5e-41	40.80%	539	gi|1126299828
gid|76|Allergen allergen Ana 0 2	*Anacardium occidentale*	61.30%	380 of 444	9.9e-70	42.70%	510	gi|25991543
gid|1572|Putative Chain A	*Prunus dulcis*	59.30%	417 of 444	6.5e-53	45.20%	524	gi|258588247
gid|392|Allergen Cor a 9 allergen	*Corylus avellana*	59.30%	390 of 444	3.4e-41	43.70%	545	gi|557792009
gid|1572|Putative prunin 1 precursor	*Prunus dulcis*	59.30%	436 of 444	2.6e-53	44.90%	543	gi|307159112
gid|160|Allergen HMW glutenin	*Triticum aestivum*	57.52%	76 of 444	4.2e-9	31.30%	307	gi|21751
gid|1572|Putative Pru du 6 allergen	*Prunus dulcis*	57.50%	168 of 444	4.8e-20	46.20%	208	gi|523916668
gid|2650|Allergen 11S globulin	*Actinidia chinensis*	56.30%	344 of 444	4.1e-67	39.70%	514	gi|82469930
gid|1093|Putative Pis v 2.0201 allergen 11S	*Pistacia vera*	56.30%	332 of 444	9.2e-53	39.50%	516	gi|110349085
gid|2274|Putative 11S globulin	*Sesamum indicum*	53.80%	345 of 444	2e-60	41.10%	482	gi|13183173
gid|574|Putative glycinin A3B4 subunit	*Glycine max*	53.80%	241 of 444	6.4e-27	32.40%	558	gi|10566449
gid|291|Allergen allergen Arah3/Arah4	*Arachis hypogaea*	47.50%	208 of 444	1.2e-20	32.40%	561	gi|21314465
**C3S7F1: Oleosin S2-2 [Brassica napus (Rape)]**							
gid|2298|Allergen oleosin	*Corylus avellana*	70.01%	109 of 109	2.2e-42	52.50%	158	gi|49617323
gid|2283|Putative oleosin 1	*Arachis hypogaea*	64.98%	104 of 109	6.5e-39	46.20%	171	gi|113200509
gid|1893|Putative oleosin	*Sesamum indicum*	62.50%	103 of 109	2.2e-31	45.90%	157	gi|10834827
gid|389|Putative oleosin	*Corylus avellana*	50.00%	68 of 109	1.1e-21	41.00%	117	gi|29170509
gid|2285|Allergen oleosin 3	*Arachis hypogaea*	45.10%	77 of 109	9.1e-21	38.20%	144	gi|52001241
**C3S7F8: Oleosin S3-1 [Brassica napus (Rape]**							
gid|1238|Putative 15 kDa oleosin	*Sesamum indicum*	76.20%	101 of 101	6e-31	63.20%	125	gi|5381321
gid|389|Putative oleosin	*Corylus avellana*	73.80%	98 of 101	1.1e-32	66.90%	121	gi|29170509
gid|2284|Putative oleosin 1	*Arachis hypogaea*	70.00%	94 of 101	1.2e-28	60.80%	125	gi|71040655
**Q6W7E8: Enolase [Brassica campestris (Field mustard)]**							
gid|586|Putative Enolase	*Hevea brasiliensis*	98.80%	365 of 365	1.1e-170	89.60%	442	gi|14423687
gid|1955|Allergen Alpha-enolase	*Thunnus albacares*	88.70%	365 of 365	2.9e-129	69.50%	442	gi|576011129
gid|1959|Allergen enolase	*Salmo salar*	88.70%	365 of 365	5.1e-130	69.90%	442	gi|385145180
gid|2710|Allergen beta-enolase	*Gallus gallus*	83.80%	365 of 365	3.6e-119	65.10%	441	gi|46048765
gid|396|Allergen Enolase	*Candida albicans*	82.50%	365 of 365	1.5e-111	63.10%	444	gi|232054
gid|103|Putative enolase	*Rhodotorula mucilaginosa*	80.00%	365 of 365	9.5e-107	61.00%	441	gi|30314940
**Q7XZT2-BRAJU Glutathione S-transferase 3 [Brassica juncea]**							
gid|856|Allergen glutathione S transferase c	*Periplaneta americana*	40.00%	14 of 134	1.2e-6	29.40%	163	gi|359326557

**^a^** gid: allergen group id number in the AllergenOnline database, which links to detailed information on the allergenicity references for the group, the type of allergen, other sequences belonging to the same group, and more on the allergenonline.org website. **^b^** Highest scoring identity for FASTA3 alignments of every possible 80 amino acid segment. The Food and Agriculture Organization/World Health Organization (FAO/WHO) 2001 expert panel recommended using a criteria of >35% identity over any segment of 80 or more amino acids as an indication of possible cross-reactivity for allergens, which was adopted by the Codex Alimentarius Commission (2003). **^c^** The E-value (expectation value) is a calculated value that reflects the degree of similarity of the query protein to its corresponding matches. The size of the E-value is inversely related to similarity of two proteins, meaning a very low E-value (e.g., 10e-30) indicates a high degree of similarity between the query sequence and the matching sequence from the database, while a value of 1 or higher indicates the proteins are not likely to be related in evolution or structure. **^d^** Overall percent identity (ID) (percentage of amino acids with a direct match in the alignment). **^e^** Length of amino sequence alignment. **^f^**Link to the unique assigned protein identity (gi number) in the NCBI (National Centre for Biotechnology Information) Protein Database.

## Supplementary Materials

The following are available online at https://www.mdpi.com/2218-273X/9/9/489/s1.
