# Supplementary material for: Detection and Identification of Allergens from Canadian Mustard Varieties of Sinapis alba and Brassica juncea
Source: Biomolecules. 2019 Sep 14;9(9):489. doi: 10.3390/biom9090489 (PMC6770452; doi:10.3390/biom9090489)
Supplement: Supplementary file 1 [file biomolecules-09-00489-s001.pdf]

# Appendix 1

## AllergenOnline Database v19 (February 10, 2019)

## FASTA 36. 80mer Sliding Window Search Results

| **P15322: Allergen Sin a 1 [Sinapis alba (White mustard)]** | |
|---|---|
| Database | AllergenOnline Database v19 (February 10, 2019) |
| **Input Query** | >query<br>PAGPFRIPKCRKEFQQAQHLRACQQWLHKQAMQSGSGPSWTLDDEFDFEDDMENPQGPQQ<br>RPPLLQQCCNELHQEEPLCVCPTLKGASKAVKQQVRQQLGQQGQQGPHLQHVISRIYQTA<br>THLPKVCNIRQVSVCPFKKTMPGPS |
| **Length** | 145 |
| **Number of 80 mers** | 66 |
| **Number of Sequences with hits** | 14 |

| Hit # | Defline | Species | Best %ID | # Hits > 35% | Full Alignment | | | Links | |
|---|---|---|---|---|---|---|---|---|---|
| | | | | | E-val | %ID | length | NCBI | Details |
| 1 | gi\|51338758\|gid\|192\|Allergen Allergen Sin a 1 precursor ( | Sinapis alba | 100.00% | 66of66 | 2.4e-034 | 100.00% | 145 | gi\|51338758 | GO! |
| 2 | gi\|1009434\|gid\|192\|Allergen allergen sin a 1.0104 [Sinapi | Sinapis alba | 100.00% | 66of66 | 2.3e-033 | 97.20% | 145 | gi\|1009434 | GO! |
| 3 | gi\|1009440\|gid\|192\|Allergen allergen sin a 1.0107 [Sinapi | Sinapis alba | 100.00% | 66of66 | 1.3e-033 | 98.60% | 145 | gi\|1009440 | GO! |
| 4 | gi\|1009438\|gid\|192\|Allergen allergen sin a 1.0106 [Sinapi | Sinapis alba | 100.00% | 66of66 | 3.5e-033 | 97.20% | 145 | gi\|1009438 | GO! |
| 5 | gi\|1009442\|gid\|192\|Allergen allergen sin a 1.0108 [Sinapi | Sinapis alba | 98.80% | 66of66 | 4.9e-033 | 96.60% | 145 | gi\|1009442 | GO! |
| 6 | gi\|1009436\|gid\|192\|Allergen allergen sin a 1.0105 [Sinapi | Sinapis alba | 98.80% | 66of66 | 4.5e-033 | 97.20% | 145 | gi\|1009436 | GO! |
| 7 | gi\|17697\|gid\|1172\|Putative 2S storage protein [Brassica r | Brassica rapa | 93.80% | 66of66 | 2.5e-029 | 88.30% | 145 | gi\|17697 | GO! |
| 8 | gi\|75107016\|gid\|1142\|Putative Napin-3 (Napin BnIII) (Napi | Brassica napus | 91.20% | 66of66 | 2.6e-017 | 82.60% | 144 | gi\|75107016 | GO! |

| | | | | | | | | | |
|---|---|---|---|---|---|---|---|---|---|
| 9 | gi\|32363444\|gid\|1170\|Putative Allergen Bra j 1-E (Bra j I | Brassica juncea | 87.50% | 66of66 | 1.8e-017 | 79.90% | 144 | gi\|32363444 | GO! |
| 10 | gi\|26985163\|gid\|386\|Putative recombinant Ib pronapin prec | Brassica napus | 53.80% | 66of66 | 0.00026 | 50.40% | 129 | gi\|26985163 | GO! |
| 11 | gi\|110349081\|gid\|1270\|Putative Pis v 1 allergen 2S albumi | Pistacia vera | 39.99% | 19of66 | 0.31 | 32.60% | 129 | gi\|110349081 | GO! |
| 12 | gi\|13183175\|gid\|204\|Putative 2S albumin [Sesamum indicum] | Sesamum indicum | 35.80% | 2of66 | 0.37 | 32.30% | 130 | gi\|13183175 | GO! |
| 13 | gi\|209165427\|gid\|204\|Putative 2S albumin [Sesamum indicum | Sesamum indicum | 35.80% | 2of66 | 0.56 | 32.30% | 130 | gi\|209165427 | GO! |
| 14 | gi\|62550933\|gid\|154\|Putative putative LMW-glutenin subuni | Triticum aestivum | 35.40% | 2of66 | 6.9 | 27.90% | 140 | gi\|62550933 | GO! |

| **P80207: Allergen Bra j 1-E [Brassica juncea (Indian mustard)]** | |
|---|---|
| **Database** | AllergenOnline Database v19 (February 10, 2019) |
| **Input Query** | >query<br>AGPFRFPRCRKEFQQAQHLRACQQWLHKQAMQSGSGPQPQGPQQRPPLLQQCCNELHQEE<br>PLCVCPTLKGASKAVKQQIRQQGQQQGQQGQQLQHEISRIYQTATHLPRVCNIPRVSICP<br>FQKTMPGPS |
| **Length** | 129 |
| **Number of 80 mers** | 50 |
| **Number of Sequences with hits** | 35 |

| Hit # | Define | Species | Best %ID | # Hits > 35% | Full Alignment | | | Links | |
|---|---|---|---|---|---|---|---|---|---|
| | | | | | E-val | %ID | length | NCBI | Details |
| 1 | gi\|32363444\|gid\|1170\|Putative Allergen Bra j 1-E (Bra j I | Brassica juncea | 100.00% | 50of50 | 7.1e-023 | 100.00% | 129 | gi\|32363444 | GO! |
| 2 | gi\|75107016\|gid\|1142\|Putative Napin-3 (Napin BnIII) (Napi | Brassica napus | 92.50% | 50of50 | 8.9e-020 | 89.10% | 129 | gi\|75107016 | GO! |
| 3 | gi\|1009434\|gid\|192\|Allergen allergen sin a 1.0104 [Sinapi | Sinapis alba | 88.78% | 50of50 | 2.5e-013 | 80.60% | 144 | gi\|1009434 | GO! |
| 4 | gi\|1009440\|gid\|192\|Allergen allergen sin a 1.0107 [Sinapi | Sinapis alba | 88.78% | 50of50 | 2e-013 | 80.60% | 144 | gi\|1009440 | GO! |

| | | | | | | | | | | |
|---|---|---|---|---|---|---|---|---|---|---|
| 5 | gi\|1009438\|gid\|192\|Allergen allergen sin a 1.0106 [Sinapi | Sinapis alba | 88.78% | 50of50 | 2.2e-013 | 79.90% | 144 | gi\|1009438 | GO! |
| 6 | gi\|1009436\|gid\|192\|Allergen allergen sin a 1.0105 [Sinapi | Sinapis alba | 88.78% | 50of50 | 2e-013 | 80.60% | 144 | gi\|1009436 | GO! |
| 7 | gi\|1009442\|gid\|192\|Allergen allergen sin a 1.0108 [Sinapi | Sinapis alba | 87.50% | 50of50 | 2.9e-013 | 79.20% | 144 | gi\|1009442 | GO! |
| 8 | gi\|51338758\|gid\|192\|Allergen Allergen Sin a 1 precursor ( | Sinapis alba | 87.50% | 50of50 | 5.7e-013 | 79.90% | 144 | gi\|51338758 | GO! |
| 9 | gi\|17697\|gid\|1172\|Putative 2S storage protein [Brassica r | Brassica rapa | 85.00% | 50of50 | 1e-011 | 77.10% | 144 | gi\|17697 | GO! |
| 10 | gi\|26985163\|gid\|386\|Putative recombinant Ib pronapin prec | Brassica napus | 55.00% | 50of50 | 3.8e-006 | 50.00% | 114 | gi\|26985163 | GO! |
| 11 | gi\|21068\|gid\|104\|Allergen 2S albumin precursor [Ricinus c | Ricinus communis | 40.00% | 11of50 | 0.16 | 34.50% | 113 | gi\|21068 | GO! |
| 12 | gi\|110349081\|gid\|1270\|Putative Pis v 1 allergen 2S albumi | Pistacia vera | 38.71% | 17of50 | 0.76 | 33.90% | 115 | gi\|110349081 | GO! |
| 13 | gi\|170708\|gid\|152\|Allergen gamma-gliadin B precursor | Triticum aestivum | 37.80% | 11of50 | 10 | 25.90% | 135 | gi\|170708 | GO! |
| 14 | gi\|209165427\|gid\|204\|Putative 2S albumin [Sesamum indicum | Sesamum indicum | 37.50% | 6of50 | 0.12 | 33.60% | 122 | gi\|209165427 | GO! |
| 15 | gi\|13183175\|gid\|204\|Putative 2S albumin [Sesamum indicum] | Sesamum indicum | 37.50% | 6of50 | 0.091 | 33.60% | 122 | gi\|13183175 | GO! |
| 16 | gi\|736319\|gid\|160\|Allergen glutenin [Triticum aestivum] | Triticum aestivum | 37.30% | 2of50 | 12 | 32.70% | 107 | gi\|736319 | GO! |
| 17 | gi\|288860106\|gid\|160\|Allergen high molecular weight glute | Triticum aestivum | 37.00% | 5of50 | 20 | 36.80% | 87 | gi\|288860106 | GO! |
| 18 | gi\|75219081\|gid\|154\|Putative LMM glutenin 1 | Triticum aestivum | 36.90% | 7of50 | 8.4 | 32.30% | 99 | gi\|75219081 | GO! |
| 19 | gi\|170736\|gid\|152\|Allergen gamma-gliadin | Triticum aestivum | 36.80% | 10of50 | 6.9 | 33.00% | 103 | gi\|170736 | GO! |
| 20 | gi\|508732621\|gid\|152\|Allergen gamma-gliadin, partial [Tri | Triticum aestivum | 36.80% | 10of50 | 7.3 | 33.00% | 103 | gi\|508732621 | GO! |

| | | | | | | | | | |
|---|---|---|---|---|---|---|---|---|---|
| 21 | gi\|886967\|gid\|154\|Putative low molecular weight glutenin | Triticum aestivum | 36.70% | 7of50 | 21 | 36.00% | 100 | gi\|886967 | GO! |
| 22 | gi\|508732623\|gid\|150\|Allergen omega-gliadin, partial [Tri | Triticum aestivum | 36.60% | 3of50 | 7.6 | 35.50% | 93 | gi\|508732623 | GO! |
| 23 | gi\|73912496\|gid\|150\|Allergen omega-5 gliadin [Triticum ae | Triticum aestivum | 36.50% | 9of50 | 7.5 | 25.40% | 122 | gi\|73912496 | GO! |
| 24 | gi\|21743\|gid\|160\|Allergen high molecular weight glutenin | Triticum aestivum | 36.40% | 3of50 | 7.6 | 36.40% | 88 | gi\|21743 | GO! |
| 25 | gi\|508732625\|gid\|160\|Allergen high molecular weight glute | Triticum aestivum | 36.40% | 2of50 | 7.4 | 36.40% | 88 | gi\|508732625 | GO! |
| 26 | gi\|886965\|gid\|154\|Putative low molecular weight glutenin | Triticum aestivum | 36.40% | 3of50 | 15 | 36.50% | 96 | gi\|886965 | GO! |
| 27 | gi\|170743\|gid\|160\|Allergen HMW glutenin subunit Ax2* [Tri | Triticum aestivum | 36.40% | 2of50 | 7.5 | 36.40% | 88 | gi\|170743 | GO! |
| 28 | gi\|886963\|gid\|154\|Putative low molecular weight glutenin | Triticum aestivum | 36.26% | 8of50 | 8.3 | 31.00% | 116 | gi\|886963 | GO! |
| 29 | gi\|170738\|gid\|152\|Allergen gamma-gliadin | Triticum aestivum | 36.24% | 6of50 | 3.6 | 27.60% | 127 | gi\|170738 | GO! |
| 30 | gi\|226437844\|gid\|1383\|Allergen 2S albumin [Corylus avella | Corylus avellana | 36.24% | 2of50 | 0.054 | 33.00% | 109 | gi\|226437844 | GO! |
| 31 | gi\|283476402\|gid\|1617\|Putative alpha/beta gliadin precurs | Triticum aestivum | 35.90% | 4of50 | 11 | 29.90% | 117 | gi\|283476402 | GO! |
| 32 | gi\|208605348\|gid\|150\|Allergen D-type LMW glutenin subunit | Triticum aestivum | 35.60% | 1of50 | 9.5 | 28.70% | 129 | gi\|208605348 | GO! |
| 33 | gi\|208605346\|gid\|150\|Allergen D-type LMW glutenin subunit | Triticum aestivum | 35.60% | 1of50 | 1 | 28.50% | 130 | gi\|208605346 | GO! |
| 34 | gi\|75317968\|gid\|154\|Putative LMM glutenin 3 | Triticum aestivum | 35.03% | 1of50 | 6 | 31.90% | 94 | gi\|75317968 | GO! |
| 35 | gi\|208605344\|gid\|150\|Allergen D-type LMW glutenin subunit | Triticum aestivum | 35.00% | 2of50 | 5.2 | 31.20% | 125 | gi\|208605344 | GO! |

| Q2TLWO: 11S globulin [Sinapis alba (White mustard)] | |
|---|---|
| **Database** | AllergenOnline Database v19 (February 10, 2019) |
| **Input Query** | >query<br>MVKLAHLLVATVGVLLVLNGCLARQSLGVPPQVKDACNLDNLDVLQPTEVIKSEAGQVEY<br>WDHNHPQIRCAGVSIARLVIQKGGLYLPTFFSSPFISYVVQGMGISGRVIPGCAETFMDS<br>QPMQGQQGQQQGQQGQQGQQGQQGQQGQQGQQGQQGQQGQQGQQGQQGQQGQGFRDMHQK<br>VEHVRHGDAIAMTPGSAQWIYNTGDQPLIIVSLIDIANYQNQLDRNPRTFRLAGNNQQGS<br>SQQQQQQQQNILSGFDPQVLAQALKIDVRLAQELQNQQDKRGNIVRVKGPFQVVRPPLRQ<br>AYESEQWRHPRGPPQSPQDNGLEETICSMRTHENIDDPARADIYKPNLGRVTSVNSYTLP<br>ILQYIRLSATRGILQGSAMVLPKYNMNANEILYCTQGQARIQVVNDNGQNVLDQQVQKGQ<br>LVVIPQGFAYVVQSQNNFEWISFKTNANAMISTLAGRTSALRALPLEVITNAYQISLEEA<br>RKIKFNTLETTLTRARGGQQPQLIEEIVEV |
| **Length** | 510 |
| **Number of 80 mers** | 431 |
| **Number of Sequences with hits** | 90 |

| Hit # | Define | Species | Best %ID | # Hits > 35% | Full Alignment | | | Links | |
|---|---|---|---|---|---|---|---|---|---|
| | | | | | E-val | %ID | length | NCBI | Details |
| 1 | gi\|62240392\|gid\|837\|Allergen 11S globulin precursor [Sina | Sinapis alba | 100.00% | 431of431 | 6.6e-160 | 92.20% | 523 | gi\|62240392 | GO! |
| 2 | gi\|62240390\|gid\|837\|Allergen 11S globulin precursor [Sina | Sinapis alba | 100.00% | 431of431 | 0 | 100.00% | 510 | gi\|62240390 | GO! |
| 3 | gi\|158998782\|gid\|2066\|Putative 11S legumin protein [Carya | Carya illinoinensis | 62.51% | 351of431 | 2.6e-061 | 43.20% | 521 | gi\|158998782 | GO! |
| 4 | gi\|1126299828\|gid\|2597\|Putative legumin [Juglans nigra] | Juglans nigra | 62.51% | 362of431 | 2.7e-058 | 42.00% | 528 | gi\|1126299828 | GO! |
| 5 | gi\|158998780\|gid\|2066\|Putative 11S legumin protein [Carya | Carya illinoinensis | 61.30% | 348of431 | 3.5e-061 | 42.80% | 521 | gi\|158998780 | GO! |
| 6 | gi\|56788031\|gid\|817\|Putative seed storage protein [Juglan | Juglans regia | 61.30% | 363of431 | 1.2e-061 | 42.30% | 534 | gi\|56788031 | GO! |

| | | | | | | | | | | |
|---|---|---|---|---|---|---|---|---|---|---|
| 7 | gi\|258588247\|gid\|1572\|Putative Chain A, Crystal Structure | Prunus dulcis | 60.50% | 424of431 | 2.1e-051 | 46.70% | 522 | gi\|258588247 | GO! |
| 8 | gi\|307159112\|gid\|1572\|Putative prunin 1 precursor [Prunus | Prunus dulcis | 60.50% | 431of431 | 8.3e-052 | 46.40% | 541 | gi\|307159112 | GO! |
| 9 | gi\|25991543\|gid\|76\|Allergen allergen Ana 0 2 [Anacardium | Anacardium occidentale | 60.04% | 380of431 | 2.3e-070 | 44.80% | 498 | gi\|25991543 | GO! |
| 10 | gi\|288860106\|gid\|160\|Allergen high molecular weight glute | Triticum aestivum | 60.00% | 84of431 | 3.5e-018 | 37.60% | 213 | gi\|288860106 | GO! |
| 11 | gi\|736319\|gid\|160\|Allergen glutenin [Triticum aestivum] | Triticum aestivum | 58.50% | 79of431 | 1.9e-013 | 35.30% | 215 | gi\|736319 | GO! |
| 12 | gi\|18479082\|gid\|392\|Allergen 11S globulin-like protein [C | Corylus avellana | 58.00% | 387of431 | 3.6e-061 | 45.70% | 534 | gi\|18479082 | GO! |
| 13 | gi\|557792009\|gid\|392\|Allergen Cor a 9 allergen [Corylus a | Corylus avellana | 58.00% | 380of431 | 1.6e-061 | 44.90% | 534 | gi\|557792009 | GO! |
| 14 | gi\|307159114\|gid\|1572\|Putative prunin 2 precursor, partia | Prunus dulcis | 57.80% | 418of431 | 3e-061 | 45.60% | 522 | gi\|307159114 | GO! |
| 15 | gi\|82469930\|gid\|2650\|Allergen 11S globulin-like protein [ | Actinidia chinensis | 57.50% | 352of431 | 2.3e-068 | 41.40% | 503 | gi\|82469930 | GO! |
| 16 | gi\|523916668\|gid\|1572\|Putative putative Pru du 6 allergen | Prunus dulcis | 56.26% | 165of431 | 8.7e-020 | 47.20% | 195 | gi\|523916668 | GO! |
| 17 | gi\|13183173\|gid\|2274\|Putative 11S globulin [Sesamum indic | Sesamum indicum | 55.00% | 342of431 | 3.7e-060 | 42.10% | 470 | gi\|13183173 | GO! |
| 18 | gi\|156001070\|gid\|1093\|Putative 11S globulin [Pistacia ver | Pistacia vera | 55.00% | 356of431 | 6.5e-053 | 41.80% | 505 | gi\|156001070 | GO! |
| 19 | gi\|110349083\|gid\|1093\|Putative Pis v 2.0101 allergen11S g | Pistacia vera | 55.00% | 346of431 | 9.9e-050 | 41.40% | 510 | gi\|110349083 | GO! |
| 20 | gi\|110349085\|gid\|1093\|Putative Pis v 2.0201 allergen 11S | Pistacia vera | 55.00% | 356of431 | 1.2e-052 | 41.60% | 505 | gi\|110349085 | GO! |
| 21 | gi\|113200131\|gid\|531\|Putative allergenic protein [Fagopyr | Fagopyrum tataricum | 55.00% | 256of431 | 1.5e-043 | 38.10% | 486 | gi\|113200131 | GO! |
| 22 | gi\|18639\|gid\|733\|Putative glycinin subunit G3 [Glycine ma | Glycine max | 53.10% | 272of431 | 2e-038 | 36.20% | 514 | gi\|18639 | GO! |
| 23 | gi\|169973\|gid\|572\|Putative glycinin A-1a-B-x subunit [Gly | Glycine max | 52.50% | 259of431 | 6.7e-044 | 37.30% | 518 | gi\|169973 | GO! |

| | | | | | | | | | | |
|---|---|---|---|---|---|---|---|---|---|---|
| 24 | gi\|18615\|gid\|572\|Putative Glycinin-like [Glycine max] | Glycine max | 52.50% | 259of431 | 3.6e-044 | 37.30% | 518 | gi\|18615 | GO! |
| 25 | gi\|30313867\|gid\|347\|Putative 11S globulin [Bertholletia e | Bertholletia excelsa | 52.50% | 318of431 | 5.8e-059 | 39.20% | 510 | gi\|30313867 | GO! |
| 26 | gi\|29839255\|gid\|531\|Putative 13S globulin seed storage pr | Fagopyrum esculentum | 51.20% | 229of431 | 1.3e-040 | 36.10% | 477 | gi\|29839255 | GO! |
| 27 | gi\|21779\|gid\|160\|Allergen HMW glutenin-like protein produ | Triticum aestivum | 50.60% | 76of431 | 2.8e-008 | 27.80% | 410 | gi\|21779 | GO! |
| 28 | gi\|18609\|gid\|732\|Putative Glycinin A2B1a subunit-like [Gl | Glycine max | 50.00% | 234of431 | 1.1e-028 | 35.70% | 504 | gi\|18609 | GO! |
| 29 | gi\|29839254\|gid\|531\|Putative 13S globulin seed storage pr | Fagopyrum esculentum | 50.00% | 246of431 | 1e-041 | 35.60% | 506 | gi\|29839254 | GO! |
| 30 | gi\|218265\|gid\|732\|Putative glycinin A2B1a subunit [Glycin | Glycine max | 50.00% | 234of431 | 1.1e-028 | 35.70% | 504 | gi\|218265 | GO! |
| 31 | gi\|29839419\|gid\|531\|Putative 13S globulin seed storage pr | Fagopyrum esculentum | 50.00% | 248of431 | 1.2e-034 | 37.00% | 487 | gi\|29839419 | GO! |
| 32 | gi\|71084277\|gid\|160\|Allergen HMW glutenin x-type subunit | Triticum aestivum | 50.00% | 83of431 | 7.2e-011 | 35.20% | 250 | gi\|71084277 | GO! |
| 33 | gi\|3703107\|gid\|291\|Allergen glycinin [Arachis hypogaea] | Arachis hypogaea | 49.40% | 173of431 | 2.7e-025 | 32.70% | 513 | gi\|3703107 | GO! |
| 34 | gi\|5381325\|gid\|2273\|Allergen 11S globulin precursor [Sesa | Sesamum indicum | 49.40% | 225of431 | 9.2e-052 | 34.60% | 503 | gi\|5381325 | GO! |
| 35 | gi\|22135348\|gid\|291\|Allergen trypsin inhibitor [Arachis h | Arachis hypogaea | 49.40% | 80of431 | 1.8e-013 | 33.70% | 243 | gi\|22135348 | GO! |
| 36 | gi\|21314465\|gid\|291\|Allergen allergen Arah3/Arah4 [Arachi | Arachis hypogaea | 49.40% | 246of431 | 3e-013 | 33.80% | 550 | gi\|21314465 | GO! |
| 37 | gi\|10566449\|gid\|574\|Putative glycinin A3B4 subunit [Glyci | Glycine max | 48.78% | 229of431 | 2.1e-027 | 32.70% | 547 | gi\|10566449 | GO! |
| 38 | gi\|21743\|gid\|160\|Allergen high molecular weight glutenin | Triticum aestivum | 48.75% | 69of431 | 3.2e-012 | 35.40% | 229 | gi\|21743 | GO! |
| 39 | gi\|5712199\|gid\|291\|Allergen glycinin [Arachis hypogaea] | Arachis hypogaea | 48.70% | 162of431 | 2.8e-023 | 32.30% | 539 | gi\|5712199 | GO! |
| 40 | gi\|224036293\|gid\|291\|Allergen Chain A, Crystal Structure | Arachis hypogaea | 48.70% | 190of431 | 1.4e-026 | 33.10% | 513 | gi\|224036293 | GO! |

| | | | | | | | | | | |
|---|---|---|---|---|---|---|---|---|---|---|
| 41 | gi\|199732457\|gid\|291\|Allergen arachin Arah3 isoform [Arac | Arachis hypogaea | 48.70% | 190of431 | 1.4e-026 | 32.80% | 539 | gi\|199732457 | GO! |
| 42 | gi\|806556\|gid\|573\|Putative A5A4B3 subunit [Glycine soja] | Glycine soja | 48.10% | 243of431 | 4.7e-028 | 31.00% | 581 | gi\|806556 | GO! |
| 43 | gi\|18641\|gid\|573\|Putative glycinin [Glycine max] | Glycine max | 48.10% | 227of431 | 3.4e-028 | 30.50% | 581 | gi\|18641 | GO! |
| 44 | gi\|4249568\|gid\|573\|Putative glycinin [Glycine max] | Glycine max | 48.10% | 243of431 | 3.4e-028 | 31.00% | 581 | gi\|4249568 | GO! |
| 45 | gi\|22090\|gid\|160\|Allergen HMW glutenin subunit 1By9 [Trit | Triticum aestivum | 48.10% | 76of431 | 2.1e-011 | 30.80% | 318 | gi\|22090 | GO! |
| 46 | gi\|21751\|gid\|160\|Allergen high molecular weight glutenin | Triticum aestivum | 47.60% | 74of431 | 1.7e-008 | 30.30% | 297 | gi\|21751 | GO! |
| 47 | gi\|508732625\|gid\|160\|Allergen high molecular weight glute | Triticum aestivum | 47.52% | 66of431 | 1.4e-012 | 35.40% | 229 | gi\|508732625 | GO! |
| 48 | gi\|170743\|gid\|160\|Allergen HMW glutenin subunit Ax2* [Tri | Triticum aestivum | 47.52% | 66of431 | 1.4e-012 | 35.40% | 229 | gi\|170743 | GO! |
| 49 | gi\|732706\|gid\|573\|Putative Glycinin-like protein product | Glycine max | 46.90% | 221of431 | 8.8e-028 | 30.50% | 580 | gi\|732706 | GO! |
| 50 | gi\|169971\|gid\|574\|Putative glycinin precursor | Glycine max | 46.30% | 115of431 | 6.2e-027 | 38.70% | 191 | gi\|169971 | GO! |
| 51 | gi\|584592120\|gid\|2481\|Putative 13S globulin [Fagopyrum es | Fagopyrum esculentum | 46.30% | 242of431 | 5.4e-027 | 33.90% | 496 | gi\|584592120 | GO! |
| 52 | gi\|169969\|gid\|574\|Putative glycinin | Glycine max | 46.30% | 215of431 | 1.3e-015 | 32.00% | 547 | gi\|169969 | GO! |
| 53 | gi\|584592116\|gid\|2481\|Putative 13S globulin [Fagopyrum es | Fagopyrum esculentum | 46.30% | 247of431 | 5.4e-027 | 34.10% | 496 | gi\|584592116 | GO! |
| 54 | gi\|312233065\|gid\|291\|Allergen Ara h 3 allergen [Arachis h | Arachis hypogaea | 46.22% | 192of431 | 4.8e-026 | 32.70% | 517 | gi\|312233065 | GO! |
| 55 | gi\|112380623\|gid\|291\|Allergen iso-Ara h3 [Arachis hypogae | Arachis hypogaea | 46.22% | 195of431 | 1.4e-026 | 32.90% | 517 | gi\|112380623 | GO! |
| 56 | gi\|21930\|gid\|154\|Putative LMW glutenin [Triticum turgidum | Triticum turgidum subsp. durum | 43.74% | 44of431 | 1.9e-006 | 29.00% | 176 | gi\|21930 | GO! |
| 57 | gi\|170732\|gid\|154\|Putative gamma-gliadin | Triticum aestivum | 43.74% | 42of431 | 5.7e-006 | 47.90% | 73 | gi\|170732 | GO! |

| | | | | | | | | | | |
|---|---|---|---|---|---|---|---|---|---|---|
| 58 | gi\|170730\|gid\|154\|Putative pre-gamma-gliadin B-I | Triticum aestivum | 43.74% | 42of431 | 5.2e-006 | 47.90% | 73 | gi\|170730 | GO! |
| 59 | gi\|62550933\|gid\|154\|Putative putative LMW-glutenin subuni | Triticum aestivum | 43.73% | 48of431 | 0.0011 | 50.70% | 69 | gi\|62550933 | GO! |
| 60 | gi\|283476402\|gid\|1617\|Putative alpha/beta gliadin precurs | Triticum aestivum | 42.54% | 40of431 | 9.5e-005 | 43.60% | 78 | gi\|283476402 | GO! |
| 61 | gi\|21926\|gid\|154\|Putative glutenin-like protein product [ | Triticum turgidum subsp. durum | 42.50% | 40of431 | 7.4e-005 | 27.30% | 176 | gi\|21926 | GO! |
| 62 | gi\|75317968\|gid\|154\|Putative LMM glutenin 3 | Triticum aestivum | 41.25% | 18of431 | 7.3e-005 | 42.70% | 75 | gi\|75317968 | GO! |
| 63 | gi\|75219081\|gid\|154\|Putative LMM glutenin 1 | Triticum aestivum | 41.23% | 36of431 | 1.5e-005 | 27.20% | 173 | gi\|75219081 | GO! |
| 64 | gi\|208605348\|gid\|150\|Allergen D-type LMW glutenin subunit | Triticum aestivum | 40.70% | 45of431 | 2e-006 | 29.00% | 245 | gi\|208605348 | GO! |
| 65 | gi\|208605346\|gid\|150\|Allergen D-type LMW glutenin subunit | Triticum aestivum | 40.70% | 45of431 | 1.5e-006 | 30.00% | 207 | gi\|208605346 | GO! |
| 66 | gi\|897811\|gid\|160\|Allergen HMW gluten-like protein produc | Triticum aestivum | 39.98% | 47of431 | 2.3e-006 | 48.40% | 64 | gi\|897811 | GO! |
| 67 | gi\|21757\|gid\|151\|Allergen Gliadin-like protein product [T | Triticum aestivum | 39.96% | 24of431 | 0.00026 | 31.30% | 163 | gi\|21757 | GO! |
| 68 | gi\|170722\|gid\|151\|Allergen pre-alpha-/beta-gliadin A-I | Triticum aestivum | 39.50% | 22of431 | 0.0001 | 29.80% | 188 | gi\|170722 | GO! |
| 69 | gi\|170736\|gid\|152\|Allergen gamma-gliadin | Triticum aestivum | 38.80% | 29of431 | 0.0094 | 29.60% | 206 | gi\|170736 | GO! |
| 70 | gi\|508732621\|gid\|152\|Allergen gamma-gliadin, partial [Tri | Triticum aestivum | 38.80% | 29of431 | 0.0057 | 29.40% | 218 | gi\|508732621 | GO! |
| 71 | gi\|21673\|gid\|151\|Allergen Alpha/beta gliadin-like protein | Triticum aestivum | 38.78% | 30of431 | 1.3e-006 | 30.20% | 182 | gi\|21673 | GO! |
| 72 | gi\|21761\|gid\|151\|Allergen Gliadin-like protein product [T | Triticum aestivum | 37.80% | 15of431 | 0.00029 | 30.00% | 180 | gi\|21761 | GO! |
| 73 | gi\|170738\|gid\|152\|Allergen gamma-gliadin | Triticum aestivum | 37.54% | 38of431 | 0.1 | 30.90% | 178 | gi\|170738 | GO! |
| 74 | gi\|73912496\|gid\|150\|Allergen omega-5 gliadin [Triticum ae | Triticum aestivum | 37.54% | 31of431 | 2.3e-006 | 30.60% | 219 | gi\|73912496 | GO! |
| 75 | gi\|170708\|gid\|152\|Allergen gamma-gliadin B precursor | Triticum aestivum | 37.54% | 4of431 | 0.27 | 28.60% | 182 | gi\|170708 | GO! |

| | | | | | | | | | |
|---|---|---|---|---|---|---|---|---|---|
| 76 | gi\|1137166044\|gid\|151\|Allergen alpha-gliadin, partial [Tr | Triticum monococcum subsp. aegilopoides | 37.54% | 43of431 | 1.9e-006 | 30.90% | 191 | gi\|1137166044 | GO! |
| 77 | gi\|170740\|gid\|151\|Allergen gliadin | Triticum urartu | 37.50% | 25of431 | 0.0005 | 30.20% | 192 | gi\|170740 | GO! |
| 78 | gi\|21765\|gid\|151\|Allergen Gliadin-like protein product [T | Triticum aestivum | 36.29% | 28of431 | 2.5e-006 | 28.80% | 208 | gi\|21765 | GO! |
| 79 | gi\|170718\|gid\|151\|Allergen alpha/beta-gliadin precursor | Triticum aestivum | 36.29% | 28of431 | 1.8e-006 | 28.80% | 208 | gi\|170718 | GO! |
| 80 | gi\|21783\|gid\|154\|Putative LMW glutenin-like protein produ | Triticum aestivum | 36.27% | 28of431 | 9.4e-005 | 50.90% | 57 | gi\|21783 | GO! |
| 81 | gi\|21755\|gid\|151\|Allergen Alpha/beta gliadin-like protein | Triticum aestivum | 36.24% | 15of431 | 0.00016 | 28.70% | 181 | gi\|21755 | GO! |
| 82 | gi\|170720\|gid\|151\|Allergen alpha/beta-gliadin precursor [ | Triticum aestivum | 36.24% | 15of431 | 0.00016 | 28.70% | 181 | gi\|170720 | GO! |
| 83 | gi\|170702\|gid\|152\|Allergen gamma gliadin precursor | Triticum aestivum | 36.24% | 21of431 | 0.0064 | 29.10% | 151 | gi\|170702 | GO! |
| 84 | gi\|508732623\|gid\|150\|Allergen omega-gliadin, partial [Tri | Triticum aestivum | 36.22% | 32of431 | 0.16 | 27.70% | 202 | gi\|508732623 | GO! |
| 85 | gi\|170734\|gid\|154\|Putative gamma gliadin B-III | Triticum aestivum | 35.03% | 29of431 | 0.00033 | 51.90% | 54 | gi\|170734 | GO! |
| 86 | gi\|508732627\|gid\|154\|Putative low molecular weight gluten | Triticum aestivum | 35.03% | 29of431 | 0.0004 | 51.90% | 54 | gi\|508732627 | GO! |
| 87 | gi\|21773\|gid\|154\|Putative LMW glutenin-like protein produ | Triticum aestivum | 35.03% | 29of431 | 0.00044 | 51.90% | 54 | gi\|21773 | GO! |
| 88 | gi\|335331566\|gid\|154\|Putative low molecular weight gluten | Triticum aestivum | 35.03% | 1of431 | 0.0027 | 23.80% | 323 | gi\|335331566 | GO! |
| 89 | gi\|170712\|gid\|151\|Allergen pre-alpha-/beta-gliadin A-II | Triticum aestivum | 35.00% | 2of431 | 6.8e-006 | 28.40% | 176 | gi\|170712 | GO! |
| 90 | gi\|170724\|gid\|151\|Allergen pre-alpha-/beta-gliadin A-IV | Triticum aestivum | 35.00% | 2of431 | 2.7e-006 | 29.00% | 176 | gi\|170724 | GO! |

| Q7XB53: Cruciferin (Fragment) [Brassica napus (Rape)] | |
|---|---|
| **Database** | AllergenOnline Database v19 (February 10, 2019) |
| **Input Query** | >query<br>QQFPNECQLDQLNALEPSHVLKAEAGRIEVWDHHAPQLRCSGVSFVRYIIESKGLYLPSF<br>FSTAKLSFVAKGEGLMGRVVPGCAETFQDSSVFQPGGGSPFGEGQGQGQQGQGQGHQGQG<br>QGQQGQQGQQGQQSQGQGFRDMHQKVEHIRTGDTIATHPGVAQWFYNDGNQPLVIVSVLD<br>LASHQNQLDRNPRPFYLAGNNPQGQVWIEGREQQPQKNILNGFTPEVLAKAFKIDVRTAQ<br>QLQNQQDNRGNIIRVQGPFSVIRPPLRSQRPQEEVNGLEETICSARCTDNLDDPSNADVY<br>KPQLGYISTLNSYDLPILRFLRLSALRGSIRQNAMVLPQWNANANAVLYVTDGEAHVQVV<br>NDNGDRVFDGQVSQGQLLSIPQGFSVVKRATSEQFRWIEFKTNANAQINTLAGRTSVLRG<br>LPLEVISNGYQISLEEARRVKFNTIETTLTHSSGPASYGGPRKADA |
| **Length** | 466 |
| **Number of 80 mers** | 387 |
| **Number of Sequences with hits** | 57 |

| Hit # | Define | Species | Best %ID | # Hits > 35% | Full Alignment | | | Links | |
|---|---|---|---|---|---|---|---|---|---|
| | | | | | E-val | %ID | length | NCBI | Details |
| 1 | gi\|62240390\|gid\|837\|Allergen 11S globulin precursor [Sina | Sinapis alba | 76.50% | 387of387 | 3.5e-029 | 61.00% | 472 | gi\|62240390 | GO! |
| 2 | gi\|62240392\|gid\|837\|Allergen 11S globulin precursor [Sina | Sinapis alba | 74.10% | 387of387 | 1.7e-017 | 59.20% | 483 | gi\|62240392 | GO! |
| 3 | gi\|557792009\|gid\|392\|Allergen Cor a 9 allergen [Corylus a | Corylus avellana | 63.70% | 353of387 | 9.4e-022 | 44.00% | 509 | gi\|557792009 | GO! |
| 4 | gi\|18479082\|gid\|392\|Allergen 11S globulin-like protein [C | Corylus avellana | 63.70% | 373of387 | 1.7e-021 | 45.20% | 500 | gi\|18479082 | GO! |
| 5 | gi\|523916668\|gid\|1572\|Putative putative Pru du 6 allergen | Prunus dulcis | 62.51% | 160of387 | 3.2e-010 | 48.20% | 193 | gi\|523916668 | GO! |
| 6 | gi\|307159114\|gid\|1572\|Putative prunin 2 precursor, partia | Prunus dulcis | 62.51% | 387of387 | 4.3e-020 | 45.90% | 488 | gi\|307159114 | GO! |
| 7 | gi\|25991543\|gid\|76\|Allergen allergen Ana 0 2 [Anacardium | Anacardium occidentale | 61.30% | 333of387 | 1.2e-022 | 44.70% | 474 | gi\|25991543 | GO! |
| 8 | gi\|1126299828\|gid\|2597\|Putative legumin [Juglans nigra] | Juglans nigra | 58.79% | 298of387 | 3.8e-021 | 42.70% | 503 | gi\|1126299828 | GO! |

| | | | | | | | | | |
|---|---|---|---|---|---|---|---|---|---|
| 9 | gi\|158998782\|gid\|2066\|Putative 11S legumin protein [Carya | Carya illinoinensis | 58.79% | 326of387 | 3.1e-021 | 43.50% | 506 | gi\|158998782 | GO! |
| 10 | gi\|307159112\|gid\|1572\|Putative prunin 1 precursor [Prunus | Prunus dulcis | 58.79% | 373of387 | 2.5e-020 | 44.30% | 515 | gi\|307159112 | GO! |
| 11 | gi\|158998780\|gid\|2066\|Putative 11S legumin protein [Carya | Carya illinoinensis | 58.79% | 326of387 | 3.4e-021 | 43.50% | 506 | gi\|158998780 | GO! |
| 12 | gi\|258588247\|gid\|1572\|Putative Chain A, Crystal Structure | Prunus dulcis | 58.79% | 365of387 | 2.2e-020 | 44.10% | 515 | gi\|258588247 | GO! |
| 13 | gi\|56788031\|gid\|817\|Putative seed storage protein [Juglan | Juglans regia | 57.52% | 325of387 | 4.4e-021 | 42.90% | 513 | gi\|56788031 | GO! |
| 14 | gi\|110349085\|gid\|1093\|Putative Pis v 2.0201 allergen 11S | Pistacia vera | 56.30% | 347of387 | 1.7e-033 | 41.60% | 459 | gi\|110349085 | GO! |
| 15 | gi\|156001070\|gid\|1093\|Putative 11S globulin [Pistacia ver | Pistacia vera | 56.30% | 338of387 | 3.2e-033 | 41.40% | 459 | gi\|156001070 | GO! |
| 16 | gi\|82469930\|gid\|2650\|Allergen 11S globulin-like protein [ | Actinidia chinensis | 56.30% | 314of387 | 3.5e-036 | 42.90% | 466 | gi\|82469930 | GO! |
| 17 | gi\|113200131\|gid\|531\|Putative allergenic protein [Fagopyr | Fagopyrum tataricum | 55.00% | 297of387 | 5.5e-029 | 40.70% | 474 | gi\|113200131 | GO! |
| 18 | gi\|110349083\|gid\|1093\|Putative Pis v 2.0101 allergen11S g | Pistacia vera | 55.00% | 313of387 | 1.6e-025 | 40.90% | 470 | gi\|110349083 | GO! |
| 19 | gi\|29839255\|gid\|531\|Putative 13S globulin seed storage pr | Fagopyrum esculentum | 55.00% | 249of387 | 9.3e-027 | 39.50% | 466 | gi\|29839255 | GO! |
| 20 | gi\|30313867\|gid\|347\|Putative 11S globulin [Bertholletia e | Bertholletia excelsa | 53.80% | 349of387 | 1.2e-024 | 43.30% | 466 | gi\|30313867 | GO! |
| 21 | gi\|18639\|gid\|733\|Putative glycinin subunit G3 [Glycine ma | Glycine max | 53.80% | 274of387 | 4.8e-022 | 38.00% | 479 | gi\|18639 | GO! |
| 22 | gi\|29839419\|gid\|531\|Putative 13S globulin seed storage pr | Fagopyrum esculentum | 53.80% | 231of387 | 9.8e-017 | 39.30% | 476 | gi\|29839419 | GO! |
| 23 | gi\|29839254\|gid\|531\|Putative 13S globulin seed storage pr | Fagopyrum esculentum | 53.80% | 217of387 | 6.6e-017 | 37.50% | 506 | gi\|29839254 | GO! |
| 24 | gi\|18615\|gid\|572\|Putative Glycinin-like [Glycine max] | Glycine max | 52.54% | 259of387 | 1.4e-016 | 37.80% | 490 | gi\|18615 | GO! |
| 25 | gi\|169973\|gid\|572\|Putative glycinin A-1a-B-x subunit [Gly | Glycine max | 52.54% | 259of387 | 1.3e-016 | 37.80% | 490 | gi\|169973 | GO! |

| | | | | | | | | | |
|---|---|---|---|---|---|---|---|---|---|
| 26 | gi\|806556\|gid\|573\|Putative A5A4B3 subunit [Glycine soja] | Glycine soja | 52.50% | 230of387 | 3.5e-014 | 32.00% | 569 | gi\|806556 | GO! |
| 27 | gi\|10566449\|gid\|574\|Putative glycinin A3B4 subunit [Glyci | Glycine max | 52.50% | 231of387 | 4.8e-013 | 34.40% | 514 | gi\|10566449 | GO! |
| 28 | gi\|4249568\|gid\|573\|Putative glycinin [Glycine max] | Glycine max | 52.50% | 230of387 | 4.6e-014 | 32.30% | 569 | gi\|4249568 | GO! |
| 29 | gi\|71084277\|gid\|160\|Allergen HMW glutenin x-type subunit | Triticum aestivum | 52.50% | 65of387 | 0.97 | 32.80% | 198 | gi\|71084277 | GO! |
| 30 | gi\|732706\|gid\|573\|Putative Glycinin-like protein product | Glycine max | 52.50% | 213of387 | 1.1e-014 | 31.90% | 568 | gi\|732706 | GO! |
| 31 | gi\|18641\|gid\|573\|Putative glycinin [Glycine max] | Glycine max | 52.50% | 230of387 | 4.6e-014 | 32.20% | 569 | gi\|18641 | GO! |
| 32 | gi\|169971\|gid\|574\|Putative glycinin precursor | Glycine max | 52.50% | 120of387 | 4e-013 | 39.90% | 188 | gi\|169971 | GO! |
| 33 | gi\|13183173\|gid\|2274\|Putative 11S globulin [Sesamum indic | Sesamum indicum | 52.46% | 318of387 | 1.6e-024 | 40.80% | 476 | gi\|13183173 | GO! |
| 34 | gi\|218265\|gid\|732\|Putative glycinin A2B1a subunit [Glycin | Glycine max | 51.29% | 215of387 | 3.4e-022 | 35.90% | 485 | gi\|218265 | GO! |
| 35 | gi\|18609\|gid\|732\|Putative Glycinin A2B1a subunit-like [Gl | Glycine max | 51.29% | 215of387 | 3.4e-022 | 35.90% | 485 | gi\|18609 | GO! |
| 36 | gi\|288860106\|gid\|160\|Allergen high molecular weight glute | Triticum aestivum | 50.00% | 74of387 | 0.0066 | 32.40% | 225 | gi\|288860106 | GO! |
| 37 | gi\|3703107\|gid\|291\|Allergen glycinin [Arachis hypogaea] | Arachis hypogaea | 49.40% | 185of387 | 4.5e-014 | 33.30% | 532 | gi\|3703107 | GO! |
| 38 | gi\|22135348\|gid\|291\|Allergen trypsin inhibitor [Arachis h | Arachis hypogaea | 49.40% | 59of387 | 1.9e-005 | 35.80% | 204 | gi\|22135348 | GO! |
| 39 | gi\|21314465\|gid\|291\|Allergen allergen Arah3/Arah4 [Arachi | Arachis hypogaea | 49.40% | 223of387 | 2.2e-014 | 34.80% | 520 | gi\|21314465 | GO! |
| 40 | gi\|736319\|gid\|160\|Allergen glutenin [Triticum aestivum] | Triticum aestivum | 48.80% | 73of387 | 0.0014 | 30.10% | 229 | gi\|736319 | GO! |
| 41 | gi\|169969\|gid\|574\|Putative glycinin | Glycine max | 48.78% | 214of387 | 1.7e-006 | 33.10% | 514 | gi\|169969 | GO! |
| 42 | gi\|312233065\|gid\|291\|Allergen Ara h 3 allergen [Arachis h | Arachis hypogaea | 48.70% | 207of387 | 1.5e-015 | 36.10% | 487 | gi\|312233065 | GO! |
| 43 | gi\|112380623\|gid\|291\|Allergen iso-Ara h3 [Arachis hypogae | Arachis hypogaea | 48.70% | 213of387 | 8.2e-016 | 36.30% | 487 | gi\|112380623 | GO! |
| 44 | gi\|5712199\|gid\|291\|Allergen glycinin [Arachis hypogaea] | Arachis hypogaea | 46.30% | 156of387 | 3.2e-013 | 32.40% | 512 | gi\|5712199 | GO! |

| | | | | | | | | | |
|---|---|---|---|---|---|---|---|---|---|
| 45 | gi\|21743\|gid\|160\|Allergen high molecular weight glutenin | Triticum aestivum | 46.29% | 73of387 | 0.0027 | 36.60% | 142 | gi\|21743 | GO! |
| 46 | gi\|199732457\|gid\|291\|Allergen arachin Arah3 isoform [Arac | Arachis hypogaea | 46.22% | 181of387 | 1.1e-014 | 33.30% | 531 | gi\|199732457 | GO! |
| 47 | gi\|224036293\|gid\|291\|Allergen Chain A, Crystal Structure | Arachis hypogaea | 46.22% | 181of387 | 1e-014 | 33.30% | 531 | gi\|224036293 | GO! |
| 48 | gi\|5381325\|gid\|2273\|Allergen 11S globulin precursor [Sesa | Sesamum indicum | 46.22% | 187of387 | 2.9e-026 | 33.80% | 453 | gi\|5381325 | GO! |
| 49 | gi\|170743\|gid\|160\|Allergen HMW glutenin subunit Ax2* [Tri | Triticum aestivum | 45.10% | 72of387 | 0.0019 | 36.60% | 142 | gi\|170743 | GO! |
| 50 | gi\|508732625\|gid\|160\|Allergen high molecular weight glute | Triticum aestivum | 45.10% | 72of387 | 0.0018 | 36.60% | 142 | gi\|508732625 | GO! |
| 51 | gi\|21751\|gid\|160\|Allergen high molecular weight glutenin | Triticum aestivum | 45.03% | 57of387 | 0.16 | 27.70% | 260 | gi\|21751 | GO! |
| 52 | gi\|22090\|gid\|160\|Allergen HMW glutenin subunit 1By9 [Trit | Triticum aestivum | 45.03% | 60of387 | 0.21 | 27.80% | 198 | gi\|22090 | GO! |
| 53 | gi\|584592116\|gid\|2481\|Putative 13S globulin [Fagopyrum es | Fagopyrum esculentum | 43.80% | 198of387 | 2.6e-024 | 34.90% | 459 | gi\|584592116 | GO! |
| 54 | gi\|584592120\|gid\|2481\|Putative 13S globulin [Fagopyrum es | Fagopyrum esculentum | 43.80% | 198of387 | 2.6e-024 | 34.90% | 459 | gi\|584592120 | GO! |
| 55 | gi\|21779\|gid\|160\|Allergen HMW glutenin-like protein produ | Triticum aestivum | 43.80% | 51of387 | 0.24 | 28.40% | 218 | gi\|21779 | GO! |
| 56 | gi\|170732\|gid\|154\|Putative gamma-gliadin | Triticum aestivum | 38.79% | 20of387 | 0.71 | 40.30% | 77 | gi\|170732 | GO! |
| 57 | gi\|170730\|gid\|154\|Putative pre-gamma-gliadin B-I | Triticum aestivum | 38.79% | 20of387 | 0.67 | 40.30% | 77 | gi\|170730 | GO! |

| **P33525: Cruciferin CRU4 [Brassica napus (Rape)** | |
|---|---|
| **Database** | AllergenOnline Database v19 (February 10, 2019) |
| **Input Query** | >query<br>MVKVPHLLVATFGVLLVLNGCLARQSLGVPPQLGNACNLDNLDVLQPTETIKSEAGRVEY<br>WDHNNPQIRCAGVSVSRVIIEQGGLYLPTFFSSPKISYVVQGMGISGRVVPGCAETFMDS<br>QPMQGQQQGQPWQGQQGQQGQQGQQGQQGQQGQQGQQGQQGQQGQQGQQQQGFRDMHQKV<br>EHVRHGDIIAITAGSSHWIYNTGDQPLVIICLLDIANYQNQLDRNPRTFRLAGNNPQGGS<br>QQQQQQQQNMLSGFDPQVLAQALKIDVRLAQELQNQQDSRGNIVRVKGPFQVVRPPLRQP |

| | |
|---|---|
| | YESEQWRHPRGPPQSPQDNGLEETICSMRTHENIDDPARADVYKPNLGRVTSVNSYTLPI<br>LQYIRLSATRGILQGNAMVLPKYNMNANEILYCTQGQARIQVVNDNGQNVLDQQVQKGQL<br>VVIPQGFAYVVQSHQNNFEWISFKTNANAMVSTLAGRTSALRALPLEVITNAFQISLEEA<br>RRIKFNTLETTLTRARGGQPQLIEEIVEA |
| **Length** | 509 |
| **Number of 80 mers** | 430 |
| **Number of Sequences with hits** | 86 |

| **Hit #** | **Defline** | **Species** | **Best %ID** | **# Hits > 35%** | **Full Alignment** | | | **Links** | |
|---|---|---|---|---|---|---|---|---|---|
| | | | | | **E-val** | **%ID** | **length** | **NCBI** | **Details** |
| 1 | gi\|62240390\|gid\|837\|Allergen 11S globulin precursor [Sina | Sinapis alba | 98.80% | 430of430 | 2.4e-210 | 91.60% | 510 | gi\|62240390 | GO! |
| 2 | gi\|62240392\|gid\|837\|Allergen 11S globulin precursor [Sina | Sinapis alba | 97.50% | 430of430 | 2.7e-157 | 90.10% | 523 | gi\|62240392 | GO! |
| 3 | gi\|158998782\|gid\|2066\|Putative 11S legumin protein [Carya | Carya illinoinensis | 65.00% | 358of430 | 1.9e-061 | 44.70% | 535 | gi\|158998782 | GO! |
| 4 | gi\|158998780\|gid\|2066\|Putative 11S legumin protein [Carya | Carya illinoinensis | 65.00% | 355of430 | 7.2e-062 | 44.50% | 535 | gi\|158998780 | GO! |
| 5 | gi\|1126299828\|gid\|2597\|Putative legumin [Juglans nigra] | Juglans nigra | 63.79% | 373of430 | 4.1e-061 | 43.30% | 533 | gi\|1126299828 | GO! |
| 6 | gi\|56788031\|gid\|817\|Putative seed storage protein [Juglan | Juglans regia | 63.79% | 368of430 | 1e-063 | 44.50% | 533 | gi\|56788031 | GO! |
| 7 | gi\|523916668\|gid\|1572\|Putative putative Pru du 6 allergen | Prunus dulcis | 60.04% | 168of430 | 4.6e-020 | 49.70% | 195 | gi\|523916668 | GO! |
| 8 | gi\|25991543\|gid\|76\|Allergen allergen Ana 0 2 [Anacardium | Anacardium occidentale | 60.04% | 379of430 | 7.9e-073 | 46.20% | 487 | gi\|25991543 | GO! |
| 9 | gi\|307159114\|gid\|1572\|Putative prunin 2 precursor, partia | Prunus dulcis | 60.04% | 425of430 | 5.6e-040 | 47.00% | 513 | gi\|307159114 | GO! |
| 10 | gi\|18479082\|gid\|392\|Allergen 11S globulin-like protein [C | Corylus avellana | 58.79% | 394of430 | 7.4e-062 | 45.60% | 528 | gi\|18479082 | GO! |
| 11 | gi\|557792009\|gid\|392\|Allergen Cor a 9 allergen [Corylus a | Corylus avellana | 58.79% | 391of430 | 4.6e-062 | 45.50% | 528 | gi\|557792009 | GO! |

| | | | | | | | | | |
|---|---|---|---|---|---|---|---|---|---|
| 12 | gi\|258588247\|gid\|1572\|Putative Chain A, Crystal Structure | Prunus dulcis | 58.40% | 423of430 | 1e-050 | 46.30% | 521 | gi\|258588247 | GO! |
| 13 | gi\|307159112\|gid\|1572\|Putative prunin 1 precursor [Prunus | Prunus dulcis | 58.40% | 430of430 | 4e-051 | 46.10% | 536 | gi\|307159112 | GO! |
| 14 | gi\|82469930\|gid\|2650\|Allergen 11S globulin-like protein [ | Actinidia chinensis | 56.30% | 327of430 | 3.5e-069 | 41.40% | 500 | gi\|82469930 | GO! |
| 15 | gi\|736319\|gid\|160\|Allergen glutenin [Triticum aestivum] | Triticum aestivum | 56.26% | 70of430 | 2.5e-011 | 33.30% | 207 | gi\|736319 | GO! |
| 16 | gi\|288860106\|gid\|160\|Allergen high molecular weight glute | Triticum aestivum | 55.60% | 79of430 | 8e-015 | 34.80% | 224 | gi\|288860106 | GO! |
| 17 | gi\|110349083\|gid\|1093\|Putative Pis v 2.0101 allergen11S g | Pistacia vera | 55.00% | 345of430 | 6.7e-051 | 41.10% | 518 | gi\|110349083 | GO! |
| 18 | gi\|156001070\|gid\|1093\|Putative 11S globulin [Pistacia ver | Pistacia vera | 55.00% | 361of430 | 4.8e-055 | 41.90% | 513 | gi\|156001070 | GO! |
| 19 | gi\|110349085\|gid\|1093\|Putative Pis v 2.0201 allergen 11S | Pistacia vera | 55.00% | 359of430 | 9.1e-055 | 41.70% | 513 | gi\|110349085 | GO! |
| 20 | gi\|71084277\|gid\|160\|Allergen HMW glutenin x-type subunit | Triticum aestivum | 55.00% | 75of430 | 1.2e-010 | 34.10% | 252 | gi\|71084277 | GO! |
| 21 | gi\|13183173\|gid\|2274\|Putative 11S globulin [Sesamum indic | Sesamum indicum | 53.80% | 343of430 | 4.8e-063 | 42.10% | 478 | gi\|13183173 | GO! |
| 22 | gi\|113200131\|gid\|531\|Putative allergenic protein [Fagopyr | Fagopyrum tataricum | 53.80% | 255of430 | 4.4e-044 | 35.60% | 523 | gi\|113200131 | GO! |
| 23 | gi\|30313867\|gid\|347\|Putative 11S globulin [Bertholletia e | Bertholletia excelsa | 52.50% | 309of430 | 2.3e-059 | 39.70% | 506 | gi\|30313867 | GO! |
| 24 | gi\|10566449\|gid\|574\|Putative glycinin A3B4 subunit [Glyci | Glycine max | 51.25% | 264of430 | 1.6e-028 | 34.40% | 546 | gi\|10566449 | GO! |
| 25 | gi\|29839254\|gid\|531\|Putative 13S globulin seed storage pr | Fagopyrum esculentum | 51.25% | 240of430 | 1.1e-042 | 34.60% | 540 | gi\|29839254 | GO! |
| 26 | gi\|18639\|gid\|733\|Putative glycinin subunit G3 [Glycine ma | Glycine max | 51.20% | 267of430 | 3e-039 | 36.00% | 516 | gi\|18639 | GO! |
| 27 | gi\|806556\|gid\|573\|Putative A5A4B3 subunit [Glycine soja] | Glycine soja | 50.60% | 256of430 | 3.7e-029 | 31.70% | 580 | gi\|806556 | GO! |
| 28 | gi\|4249568\|gid\|573\|Putative glycinin [Glycine max] | Glycine max | 50.60% | 256of430 | 2.7e-029 | 31.70% | 580 | gi\|4249568 | GO! |

| | | | | | | | | | | |
|---|---|---|---|---|---|---|---|---|---|---|
| 29 | gi\|18641\|gid\|573\|Putative glycinin [Glycine max] | Glycine max | 50.60% | 239of430 | 2.7e-029 | 31.40% | 580 | gi\|18641 | GO! |
| 30 | gi\|22090\|gid\|160\|Allergen HMW glutenin subunit 1By9 [Trit | Triticum aestivum | 50.02% | 69of430 | 1e-010 | 30.60% | 222 | gi\|22090 | GO! |
| 31 | gi\|508732625\|gid\|160\|Allergen high molecular weight glute | Triticum aestivum | 50.00% | 70of430 | 6.7e-012 | 31.50% | 267 | gi\|508732625 | GO! |
| 32 | gi\|29839255\|gid\|531\|Putative 13S globulin seed storage pr | Fagopyrum esculentum | 50.00% | 213of430 | 3.2e-041 | 34.20% | 512 | gi\|29839255 | GO! |
| 33 | gi\|170743\|gid\|160\|Allergen HMW glutenin subunit Ax2* [Tri | Triticum aestivum | 50.00% | 70of430 | 6.9e-012 | 31.50% | 267 | gi\|170743 | GO! |
| 34 | gi\|169973\|gid\|572\|Putative glycinin A-1a-B-x subunit [Gly | Glycine max | 50.00% | 267of430 | 2.3e-046 | 37.10% | 517 | gi\|169973 | GO! |
| 35 | gi\|18615\|gid\|572\|Putative Glycinin-like [Glycine max] | Glycine max | 50.00% | 267of430 | 1.2e-046 | 37.10% | 517 | gi\|18615 | GO! |
| 36 | gi\|29839419\|gid\|531\|Putative 13S globulin seed storage pr | Fagopyrum esculentum | 50.00% | 236of430 | 2.1e-044 | 36.80% | 486 | gi\|29839419 | GO! |
| 37 | gi\|169969\|gid\|574\|Putative glycinin | Glycine max | 50.00% | 242of430 | 6.3e-015 | 33.50% | 546 | gi\|169969 | GO! |
| 38 | gi\|21743\|gid\|160\|Allergen high molecular weight glutenin | Triticum aestivum | 50.00% | 69of430 | 3.7e-012 | 31.80% | 267 | gi\|21743 | GO! |
| 39 | gi\|732706\|gid\|573\|Putative Glycinin-like protein product | Glycine max | 49.40% | 237of430 | 1.1e-028 | 31.30% | 579 | gi\|732706 | GO! |
| 40 | gi\|21751\|gid\|160\|Allergen high molecular weight glutenin | Triticum aestivum | 48.78% | 71of430 | 1.3e-009 | 30.50% | 266 | gi\|21751 | GO! |
| 41 | gi\|218265\|gid\|732\|Putative glycinin A2B1a subunit [Glycin | Glycine max | 48.70% | 244of430 | 1.4e-029 | 35.40% | 503 | gi\|218265 | GO! |
| 42 | gi\|18609\|gid\|732\|Putative Glycinin A2B1a subunit-like [Gl | Glycine max | 48.70% | 244of430 | 1.4e-029 | 35.40% | 503 | gi\|18609 | GO! |
| 43 | gi\|5381325\|gid\|2273\|Allergen 11S globulin precursor [Sesa | Sesamum indicum | 48.10% | 238of430 | 5.9e-054 | 35.00% | 503 | gi\|5381325 | GO! |
| 44 | gi\|224036293\|gid\|291\|Allergen Chain A, Crystal Structure | Arachis hypogaea | 47.50% | 187of430 | 6.1e-027 | 32.70% | 511 | gi\|224036293 | GO! |
| 45 | gi\|199732457\|gid\|291\|Allergen arachin Arah3 isoform [Arac | Arachis hypogaea | 47.50% | 187of430 | 6.4e-027 | 32.70% | 511 | gi\|199732457 | GO! |

| | | | | | | | | | |
|---|---|---|---|---|---|---|---|---|---|
| 46 | gi\|21314465\|gid\|291\|Allergen allergen Arah3/Arah4 [Arachi | Arachis hypogaea | 47.50% | 219of430 | 2.1e-025 | 33.20% | 548 | gi\|21314465 | GO! |
| 47 | gi\|169971\|gid\|574\|Putative glycinin precursor | Glycine max | 47.50% | 116of430 | 4.9e-028 | 39.80% | 191 | gi\|169971 | GO! |
| 48 | gi\|5712199\|gid\|291\|Allergen glycinin [Arachis hypogaea] | Arachis hypogaea | 47.50% | 145of430 | 1.5e-023 | 31.70% | 511 | gi\|5712199 | GO! |
| 49 | gi\|22135348\|gid\|291\|Allergen trypsin inhibitor [Arachis h | Arachis hypogaea | 47.50% | 77of430 | 3.1e-014 | 34.70% | 242 | gi\|22135348 | GO! |
| 50 | gi\|21779\|gid\|160\|Allergen HMW glutenin-like protein produ | Triticum aestivum | 47.50% | 68of430 | 1.2e-006 | 26.60% | 418 | gi\|21779 | GO! |
| 51 | gi\|3703107\|gid\|291\|Allergen glycinin [Arachis hypogaea] | Arachis hypogaea | 46.90% | 174of430 | 1.2e-025 | 32.10% | 511 | gi\|3703107 | GO! |
| 52 | gi\|584592120\|gid\|2481\|Putative 13S globulin [Fagopyrum es | Fagopyrum esculentum | 46.30% | 218of430 | 3.1e-028 | 34.10% | 496 | gi\|584592120 | GO! |
| 53 | gi\|584592116\|gid\|2481\|Putative 13S globulin [Fagopyrum es | Fagopyrum esculentum | 46.30% | 228of430 | 3.1e-028 | 34.30% | 496 | gi\|584592116 | GO! |
| 54 | gi\|112380623\|gid\|291\|Allergen iso-Ara h3 [Arachis hypogae | Arachis hypogaea | 46.22% | 186of430 | 7.2e-027 | 33.50% | 516 | gi\|112380623 | GO! |
| 55 | gi\|312233065\|gid\|291\|Allergen Ara h 3 allergen [Arachis h | Arachis hypogaea | 46.22% | 170of430 | 2.6e-026 | 33.30% | 516 | gi\|312233065 | GO! |
| 56 | gi\|21930\|gid\|154\|Putative LMW glutenin [Triticum turgidum | Triticum turgidum subsp. durum | 43.80% | 35of430 | 3e-006 | 27.50% | 171 | gi\|21930 | GO! |
| 57 | gi\|170738\|gid\|152\|Allergen gamma-gliadin | Triticum aestivum | 43.79% | 33of430 | 0.0032 | 29.80% | 208 | gi\|170738 | GO! |
| 58 | gi\|170730\|gid\|154\|Putative pre-gamma-gliadin B-I | Triticum aestivum | 43.71% | 38of430 | 3e-005 | 40.50% | 79 | gi\|170730 | GO! |
| 59 | gi\|170732\|gid\|154\|Putative gamma-gliadin | Triticum aestivum | 43.71% | 38of430 | 3.2e-005 | 40.50% | 79 | gi\|170732 | GO! |
| 60 | gi\|75219081\|gid\|154\|Putative LMM glutenin 1 | Triticum aestivum | 42.52% | 37of430 | 1.5e-005 | 26.60% | 173 | gi\|75219081 | GO! |
| 61 | gi\|62550933\|gid\|154\|Putative putative LMW-glutenin subuni | Triticum aestivum | 42.52% | 46of430 | 0.047 | 49.30% | 69 | gi\|62550933 | GO! |
| 62 | gi\|283476402\|gid\|1617\|Putative alpha/beta gliadin precurs | Triticum aestivum | 41.29% | 36of430 | 0.00054 | 25.90% | 228 | gi\|283476402 | GO! |
| 63 | gi\|170736\|gid\|152\|Allergen gamma-gliadin | Triticum aestivum | 41.28% | 35of430 | 0.015 | 32.30% | 155 | gi\|170736 | GO! |

| | | | | | | | | | |
|---|---|---|---|---|---|---|---|---|---|
| 64 | gi\|508732621\|gid\|152\|Allergen gamma-gliadin, partial [Tri | Triticum aestivum | 41.28% | 35of430 | 0.00086 | 31.00% | 203 | gi\|508732621 | GO! |
| 65 | gi\|1137166044\|gid\|151\|Allergen alpha-gliadin, partial [Tr | Triticum monococcum subsp. aegilopoides | 40.01% | 39of430 | 8.8e-007 | 29.70% | 195 | gi\|1137166044 | GO! |
| 66 | gi\|897811\|gid\|160\|Allergen HMW gluten-like protein produc | Triticum aestivum | 40.01% | 38of430 | 9.7e-006 | 48.40% | 64 | gi\|897811 | GO! |
| 67 | gi\|208605348\|gid\|150\|Allergen D-type LMW glutenin subunit | Triticum aestivum | 40.00% | 21of430 | 7.7e-005 | 27.20% | 250 | gi\|208605348 | GO! |
| 68 | gi\|208605346\|gid\|150\|Allergen D-type LMW glutenin subunit | Triticum aestivum | 40.00% | 21of430 | 6.6e-005 | 29.20% | 209 | gi\|208605346 | GO! |
| 69 | gi\|21757\|gid\|151\|Allergen Gliadin-like protein product [T | Triticum aestivum | 39.99% | 35of430 | 0.00019 | 29.80% | 178 | gi\|21757 | GO! |
| 70 | gi\|21761\|gid\|151\|Allergen Gliadin-like protein product [T | Triticum aestivum | 38.79% | 14of430 | 6.1e-005 | 26.80% | 179 | gi\|21761 | GO! |
| 71 | gi\|21926\|gid\|154\|Putative glutenin-like protein product [ | Triticum turgidum subsp. durum | 38.78% | 36of430 | 3.9e-005 | 24.10% | 174 | gi\|21926 | GO! |
| 72 | gi\|170708\|gid\|152\|Allergen gamma-gliadin B precursor | Triticum aestivum | 38.77% | 32of430 | 0.029 | 29.50% | 200 | gi\|170708 | GO! |
| 73 | gi\|170702\|gid\|152\|Allergen gamma gliadin precursor | Triticum aestivum | 38.76% | 27of430 | 0.058 | 28.20% | 188 | gi\|170702 | GO! |
| 74 | gi\|170724\|gid\|151\|Allergen pre-alpha-/beta-gliadin A-IV | Triticum aestivum | 38.76% | 18of430 | 6e-006 | 28.30% | 240 | gi\|170724 | GO! |
| 75 | gi\|73912496\|gid\|150\|Allergen omega-5 gliadin [Triticum ae | Triticum aestivum | 38.72% | 27of430 | 1.1e-006 | 29.00% | 217 | gi\|73912496 | GO! |
| 76 | gi\|1063270\|gid\|152\|Allergen gamma-gliadin precursor [Trit | Triticum aestivum | 38.72% | 1of430 | 0.024 | 30.10% | 193 | gi\|1063270 | GO! |
| 77 | gi\|208605344\|gid\|150\|Allergen D-type LMW glutenin subunit | Triticum aestivum | 37.54% | 1of430 | 0.19 | 26.40% | 231 | gi\|208605344 | GO! |
| 78 | gi\|508732623\|gid\|150\|Allergen omega-gliadin, partial [Tri | Triticum aestivum | 37.54% | 31of430 | 0.16 | 45.50% | 66 | gi\|508732623 | GO! |
| 79 | gi\|21673\|gid\|151\|Allergen Alpha/beta gliadin-like protein | Triticum aestivum | 37.52% | 30of430 | 5e-007 | 28.80% | 243 | gi\|21673 | GO! |
| 80 | gi\|170740\|gid\|151\|Allergen gliadin | Triticum urartu | 37.52% | 30of430 | 0.00049 | 29.40% | 177 | gi\|170740 | GO! |

| | | | | | | | | | | |
|---|---|---|---|---|---|---|---|---|---|---|
| 81 | gi\|21783\|gid\|154\|Putative LMW glutenin-like protein produ | Triticum aestivum | 37.00% | 3of430 | 0.00039 | 26.20% | 240 | gi\|21783 | GO! |
| 82 | gi\|75317968\|gid\|154\|Putative LMM glutenin 3 | Triticum aestivum | 36.27% | 2of430 | 0.00035 | 27.90% | 208 | gi\|75317968 | GO! |
| 83 | gi\|170726\|gid\|151\|Allergen pre-alpha-/beta-gliadin A-III | Triticum aestivum | 36.24% | 10of430 | 0.021 | 27.50% | 204 | gi\|170726 | GO! |
| 84 | gi\|21765\|gid\|151\|Allergen Gliadin-like protein product [T | Triticum aestivum | 35.04% | 3of430 | 2.1e-006 | 29.10% | 234 | gi\|21765 | GO! |
| 85 | gi\|170718\|gid\|151\|Allergen alpha/beta-gliadin precursor | Triticum aestivum | 35.04% | 3of430 | 1.5e-006 | 29.10% | 234 | gi\|170718 | GO! |
| 86 | gi\|170712\|gid\|151\|Allergen pre-alpha-/beta-gliadin A-II | Triticum aestivum | 35.02% | 15of430 | 2.1e-005 | 29.00% | 183 | gi\|170712 | GO! |

---

| Q2TLV9: 11S globulin [Sinapis alba (White mustard)] | |
|---|---|
| **Database** | AllergenOnline Database v19 (February 10, 2019) |
| **Input Query** | >query<br>MVKLAHLLVATVGVLLVLNGCLARQSLGVPPQVKDACNLDNLDVLQPTETIKSEAGRLEY<br>WDHNNPQIRCAGVSIARLVIEQGGFYLPTFFSSPKISYVVQGMGISGRVIPGCAETFMDS<br>QPMQGQQQGHQGQQQGQQGQQGQQGQQGQQGQQQGQQGQQGQQGQQGQQGQQGQQGQQQQ<br>GQQGFRDMYQKVEHVRHGDVIANTPGSAHWIYNTGDKPLVIISLLDIANYQNQLDRNPRV<br>FRLAGNNPQGGFGGPQQQQPQQNILNGFDPQVIAQALKIDVRLAQELQNKQDSRGNIVRV<br>KGPFQVVRPPLRQAYESEQWRHPRGPPQSPQDNGLEETICSMRTHENIDDPARADIYKPN<br>LGRVTSVNSYTIPILQYIRLSATRGILQGSAMVLPKYNMNANEILYCTQGQARIQVVNDN<br>GQNVLDQQVQKGQLVVIPQGFAYVVQSQNNFEWISFKTNANAMISTLAGRTSALRALPLE<br>VITNAFQISLEEARKIKFNTLETTLTRARGGQQPQLIEEIVEV |
| **Length** | 523 |
| **Number of 80 mers** | 444 |
| **Number of Sequences with hits** | 96 |

| Hit # | Defline | Species | Best %ID | # Hits > 35% | Full Alignment | | | Links | |
|---|---|---|---|---|---|---|---|---|---|
| | | | | | E-val | %ID | length | NCBI | Details |

| | | | | | | | | | | |
|---|---|---|---|---|---|---|---|---|---|---|
| 1 | gi\|62240390\|gid\|837\|Allergen 11S globulin precursor [Sina | Sinapis alba | 100.00% | 444of444 | 6.6e-160 | 92.20% | 523 | gi\|62240390 | GO! |
| 2 | gi\|62240392\|gid\|837\|Allergen 11S globulin precursor [Sina | Sinapis alba | 100.00% | 444of444 | 0 | 100.00% | 523 | gi\|62240392 | GO! |
| 3 | gi\|288860106\|gid\|160\|Allergen high molecular weight glute | Triticum aestivum | 68.80% | 99of444 | 1.3e-012 | 38.60% | 228 | gi\|288860106 | GO! |
| 4 | gi\|736319\|gid\|160\|Allergen glutenin [Triticum aestivum] | Triticum aestivum | 66.21% | 97of444 | 4.7e-014 | 35.10% | 282 | gi\|736319 | GO! |
| 5 | gi\|158998782\|gid\|2066\|Putative 11S legumin protein [Carya | Carya illinoinensis | 63.79% | 360of444 | 6.3e-041 | 41.90% | 532 | gi\|158998782 | GO! |
| 6 | gi\|56788031\|gid\|817\|Putative seed storage protein [Juglan | Juglans regia | 62.51% | 367of444 | 1.1e-041 | 41.70% | 545 | gi\|56788031 | GO! |
| 7 | gi\|1126299828\|gid\|2597\|Putative legumin [Juglans nigra] | Juglans nigra | 62.51% | 364of444 | 2.5e-041 | 40.80% | 539 | gi\|1126299828 | GO! |
| 8 | gi\|158998780\|gid\|2066\|Putative 11S legumin protein [Carya | Carya illinoinensis | 62.51% | 357of444 | 5.4e-041 | 41.50% | 532 | gi\|158998780 | GO! |
| 9 | gi\|25991543\|gid\|76\|Allergen allergen Ana 0 2 [Anacardium | Anacardium occidentale | 61.30% | 380of444 | 9.9e-070 | 42.70% | 510 | gi\|25991543 | GO! |
| 10 | gi\|18479082\|gid\|392\|Allergen 11S globulin-like protein [C | Corylus avellana | 59.30% | 399of444 | 4e-041 | 44.20% | 545 | gi\|18479082 | GO! |
| 11 | gi\|258588247\|gid\|1572\|Putative Chain A, Crystal Structure | Prunus dulcis | 59.30% | 417of444 | 6.5e-053 | 45.20% | 524 | gi\|258588247 | GO! |
| 12 | gi\|557792009\|gid\|392\|Allergen Cor a 9 allergen [Corylus a | Corylus avellana | 59.30% | 390of444 | 3.4e-041 | 43.70% | 545 | gi\|557792009 | GO! |
| 13 | gi\|307159112\|gid\|1572\|Putative prunin 1 precursor [Prunus | Prunus dulcis | 59.30% | 436of444 | 2.6e-053 | 44.90% | 543 | gi\|307159112 | GO! |
| 14 | gi\|307159114\|gid\|1572\|Putative prunin 2 precursor, partia | Prunus dulcis | 58.00% | 423of444 | 3.4e-062 | 44.00% | 534 | gi\|307159114 | GO! |
| 15 | gi\|21751\|gid\|160\|Allergen high molecular weight glutenin | Triticum aestivum | 57.52% | 76of444 | 4.2e-009 | 31.30% | 307 | gi\|21751 | GO! |
| 16 | gi\|71084277\|gid\|160\|Allergen HMW glutenin x-type subunit | Triticum aestivum | 57.52% | 85of444 | 6.8e-010 | 34.80% | 253 | gi\|71084277 | GO! |
| 17 | gi\|523916668\|gid\|1572\|Putative putative Pru du 6 allergen | Prunus dulcis | 57.50% | 168of444 | 4.8e-020 | 46.20% | 208 | gi\|523916668 | GO! |

| | | | | | | | | | |
|---|---|---|---|---|---|---|---|---|---|
| 18 | gi\|22090\|gid\|160\|Allergen HMW glutenin subunit 1By9 [Trit | Triticum aestivum | 57.48% | 84of444 | 4e-011 | 32.10% | 287 | gi\|22090 | GO! |
| 19 | gi\|21743\|gid\|160\|Allergen high molecular weight glutenin | Triticum aestivum | 56.80% | 89of444 | 2.2e-011 | 35.20% | 267 | gi\|21743 | GO! |
| 20 | gi\|21779\|gid\|160\|Allergen HMW glutenin-like protein produ | Triticum aestivum | 56.60% | 81of444 | 9.5e-009 | 28.60% | 416 | gi\|21779 | GO! |
| 21 | gi\|82469930\|gid\|2650\|Allergen 11S globulin-like protein [ | Actinidia chinensis | 56.30% | 344of444 | 4.1e-067 | 39.70% | 514 | gi\|82469930 | GO! |
| 22 | gi\|110349085\|gid\|1093\|Putative Pis v 2.0201 allergen 11S | Pistacia vera | 56.30% | 332of444 | 9.2e-053 | 39.50% | 516 | gi\|110349085 | GO! |
| 23 | gi\|156001070\|gid\|1093\|Putative 11S globulin [Pistacia ver | Pistacia vera | 56.30% | 332of444 | 4.9e-053 | 39.70% | 516 | gi\|156001070 | GO! |
| 24 | gi\|110349083\|gid\|1093\|Putative Pis v 2.0101 allergen11S g | Pistacia vera | 56.30% | 335of444 | 1.6e-047 | 39.50% | 522 | gi\|110349083 | GO! |
| 25 | gi\|508732625\|gid\|160\|Allergen high molecular weight glute | Triticum aestivum | 56.24% | 87of444 | 2.1e-011 | 34.20% | 275 | gi\|508732625 | GO! |
| 26 | gi\|170743\|gid\|160\|Allergen HMW glutenin subunit Ax2* [Tri | Triticum aestivum | 56.24% | 87of444 | 2.1e-011 | 34.20% | 275 | gi\|170743 | GO! |
| 27 | gi\|13183173\|gid\|2274\|Putative 11S globulin [Sesamum indic | Sesamum indicum | 53.80% | 345of444 | 2e-060 | 41.10% | 482 | gi\|13183173 | GO! |
| 28 | gi\|10566449\|gid\|574\|Putative glycinin A3B4 subunit [Glyci | Glycine max | 53.80% | 241of444 | 6.4e-027 | 32.40% | 558 | gi\|10566449 | GO! |
| 29 | gi\|113200131\|gid\|531\|Putative allergenic protein [Fagopyr | Fagopyrum tataricum | 53.80% | 226of444 | 2e-042 | 35.20% | 497 | gi\|113200131 | GO! |
| 30 | gi\|806556\|gid\|573\|Putative A5A4B3 subunit [Glycine soja] | Glycine soja | 52.50% | 236of444 | 8.3e-027 | 30.20% | 592 | gi\|806556 | GO! |
| 31 | gi\|169973\|gid\|572\|Putative glycinin A-1a-B-x subunit [Gly | Glycine max | 52.50% | 249of444 | 1.3e-031 | 35.70% | 529 | gi\|169973 | GO! |
| 32 | gi\|4249568\|gid\|573\|Putative glycinin [Glycine max] | Glycine max | 52.50% | 236of444 | 6.1e-027 | 30.20% | 592 | gi\|4249568 | GO! |
| 33 | gi\|18615\|gid\|572\|Putative Glycinin-like [Glycine max] | Glycine max | 52.50% | 250of444 | 1.5e-031 | 35.70% | 529 | gi\|18615 | GO! |
| 34 | gi\|18641\|gid\|573\|Putative glycinin [Glycine max] | Glycine max | 52.50% | 220of444 | 6e-027 | 29.70% | 592 | gi\|18641 | GO! |

| | | | | | | | | | |
|---|---|---|---|---|---|---|---|---|---|
| 35 | gi\|18639\|gid\|733\|Putative glycinin subunit G3 [Glycine ma | Glycine max | 51.90% | 252of444 | 3.3e-038 | 35.00% | 525 | gi\|18639 | GO! |
| 36 | gi\|169969\|gid\|574\|Putative glycinin | Glycine max | 51.20% | 224of444 | 1.9e-015 | 31.50% | 558 | gi\|169969 | GO! |
| 37 | gi\|30313867\|gid\|347\|Putative 11S globulin [Bertholletia e | Bertholletia excelsa | 51.20% | 308of444 | 8.1e-057 | 37.90% | 522 | gi\|30313867 | GO! |
| 38 | gi\|732706\|gid\|573\|Putative Glycinin-like protein product | Glycine max | 51.20% | 216of444 | 1.1e-026 | 29.80% | 591 | gi\|732706 | GO! |
| 39 | gi\|218265\|gid\|732\|Putative glycinin A2B1a subunit [Glycin | Glycine max | 50.00% | 206of444 | 7.2e-029 | 34.20% | 515 | gi\|218265 | GO! |
| 40 | gi\|18609\|gid\|732\|Putative Glycinin A2B1a subunit-like [Gl | Glycine max | 50.00% | 206of444 | 7.2e-029 | 34.20% | 515 | gi\|18609 | GO! |
| 41 | gi\|29839419\|gid\|531\|Putative 13S globulin seed storage pr | Fagopyrum esculentum | 49.40% | 221of444 | 3.1e-062 | 35.20% | 497 | gi\|29839419 | GO! |
| 42 | gi\|29839255\|gid\|531\|Putative 13S globulin seed storage pr | Fagopyrum esculentum | 49.40% | 178of444 | 4.1e-038 | 33.20% | 488 | gi\|29839255 | GO! |
| 43 | gi\|29839254\|gid\|531\|Putative 13S globulin seed storage pr | Fagopyrum esculentum | 49.40% | 223of444 | 1.3e-040 | 34.40% | 506 | gi\|29839254 | GO! |
| 44 | gi\|3703107\|gid\|291\|Allergen glycinin [Arachis hypogaea] | Arachis hypogaea | 48.78% | 152of444 | 2.8e-025 | 32.00% | 525 | gi\|3703107 | GO! |
| 45 | gi\|5381325\|gid\|2273\|Allergen 11S globulin precursor [Sesa | Sesamum indicum | 48.10% | 218of444 | 6.9e-052 | 33.30% | 514 | gi\|5381325 | GO! |
| 46 | gi\|21314465\|gid\|291\|Allergen allergen Arah3/Arah4 [Arachi | Arachis hypogaea | 47.50% | 208of444 | 1.2e-020 | 32.40% | 561 | gi\|21314465 | GO! |
| 47 | gi\|170738\|gid\|152\|Allergen gamma-gliadin | Triticum aestivum | 47.50% | 52of444 | 0.00068 | 30.90% | 207 | gi\|170738 | GO! |
| 48 | gi\|5712199\|gid\|291\|Allergen glycinin [Arachis hypogaea] | Arachis hypogaea | 47.50% | 135of444 | 8.7e-023 | 30.70% | 550 | gi\|5712199 | GO! |
| 49 | gi\|22135348\|gid\|291\|Allergen trypsin inhibitor [Arachis h | Arachis hypogaea | 47.50% | 72of444 | 1.3e-014 | 33.30% | 252 | gi\|22135348 | GO! |
| 50 | gi\|199732457\|gid\|291\|Allergen arachin Arah3 isoform [Arac | Arachis hypogaea | 47.50% | 180of444 | 4.4e-026 | 31.90% | 551 | gi\|199732457 | GO! |
| 51 | gi\|224036293\|gid\|291\|Allergen Chain A, Crystal Structure | Arachis hypogaea | 47.50% | 180of444 | 4.2e-026 | 32.20% | 525 | gi\|224036293 | GO! |

| | | | | | | | | | | |
|---|---|---|---|---|---|---|---|---|---|---|
| 52 | gi\|312233065\|gid\|291\|Allergen Ara h 3 allergen [Arachis h | Arachis hypogaea | 46.30% | 176of444 | 7.8e-038 | 31.90% | 529 | gi\|312233065 | GO! |
| 53 | gi\|73912496\|gid\|150\|Allergen omega-5 gliadin [Triticum ae | Triticum aestivum | 46.30% | 54of444 | 0.00052 | 28.60% | 259 | gi\|73912496 | GO! |
| 54 | gi\|112380623\|gid\|291\|Allergen iso-Ara h3 [Arachis hypogae | Arachis hypogaea | 46.30% | 180of444 | 2.2e-038 | 32.10% | 529 | gi\|112380623 | GO! |
| 55 | gi\|283476402\|gid\|1617\|Putative alpha/beta gliadin precurs | Triticum aestivum | 46.27% | 38of444 | 1.2e-006 | 44.90% | 78 | gi\|283476402 | GO! |
| 56 | gi\|62550933\|gid\|154\|Putative putative LMW-glutenin subuni | Triticum aestivum | 46.24% | 53of444 | 5.9e-006 | 57.80% | 64 | gi\|62550933 | GO! |
| 57 | gi\|170736\|gid\|152\|Allergen gamma-gliadin | Triticum aestivum | 45.20% | 48of444 | 0.00016 | 49.30% | 69 | gi\|170736 | GO! |
| 58 | gi\|508732621\|gid\|152\|Allergen gamma-gliadin, partial [Tri | Triticum aestivum | 45.20% | 46of444 | 6e-005 | 29.90% | 204 | gi\|508732621 | GO! |
| 59 | gi\|170708\|gid\|152\|Allergen gamma-gliadin B precursor | Triticum aestivum | 45.20% | 44of444 | 0.0005 | 30.20% | 212 | gi\|170708 | GO! |
| 60 | gi\|169971\|gid\|574\|Putative glycinin precursor | Glycine max | 45.05% | 110of444 | 1.9e-026 | 37.70% | 191 | gi\|169971 | GO! |
| 61 | gi\|208605346\|gid\|150\|Allergen D-type LMW glutenin subunit | Triticum aestivum | 45.00% | 57of444 | 0.00033 | 30.30% | 211 | gi\|208605346 | GO! |
| 62 | gi\|208605348\|gid\|150\|Allergen D-type LMW glutenin subunit | Triticum aestivum | 45.00% | 53of444 | 9.3e-005 | 28.70% | 254 | gi\|208605348 | GO! |
| 63 | gi\|170732\|gid\|154\|Putative gamma-gliadin | Triticum aestivum | 45.00% | 53of444 | 1e-006 | 45.00% | 80 | gi\|170732 | GO! |
| 64 | gi\|170730\|gid\|154\|Putative pre-gamma-gliadin B-I | Triticum aestivum | 45.00% | 53of444 | 9.5e-007 | 45.00% | 80 | gi\|170730 | GO! |
| 65 | gi\|1063270\|gid\|152\|Allergen gamma-gliadin precursor [Trit | Triticum aestivum | 44.99% | 41of444 | 4.4e-005 | 30.30% | 198 | gi\|1063270 | GO! |
| 66 | gi\|21930\|gid\|154\|Putative LMW glutenin [Triticum turgidum | Triticum turgidum subsp. durum | 44.97% | 56of444 | 4.6e-007 | 30.60% | 173 | gi\|21930 | GO! |
| 67 | gi\|508732623\|gid\|150\|Allergen omega-gliadin, partial [Tri | Triticum aestivum | 44.95% | 40of444 | 6.2e-005 | 27.10% | 251 | gi\|508732623 | GO! |
| 68 | gi\|170702\|gid\|152\|Allergen gamma gliadin precursor | Triticum aestivum | 44.80% | 36of444 | 0.0012 | 39.10% | 110 | gi\|170702 | GO! |
| 69 | gi\|584592120\|gid\|2481\|Putative 13S globulin [Fagopyrum es | Fagopyrum esculentum | 43.80% | 167of444 | 3.1e-026 | 31.70% | 508 | gi\|584592120 | GO! |

| | | | | | | | | | |
|---|---|---|---|---|---|---|---|---|---|
| 70 | gi\|584592116\|gid\|2481\|Putative 13S globulin [Fagopyrum es | Fagopyrum esculentum | 43.80% | 172of444 | 3.1e-026 | 31.90% | 508 | gi\|584592116 | GO! |
| 71 | gi\|170710\|gid\|151\|Allergen alpha-type gliadin precursor p | Triticum aestivum | 41.25% | 38of444 | 6.1e-005 | 28.70% | 244 | gi\|170710 | GO! |
| 72 | gi\|208605344\|gid\|150\|Allergen D-type LMW glutenin subunit | Triticum aestivum | 41.24% | 28of444 | 6.7e-006 | 27.70% | 242 | gi\|208605344 | GO! |
| 73 | gi\|335331566\|gid\|154\|Putative low molecular weight gluten | Triticum aestivum | 41.24% | 27of444 | 0.0045 | 22.30% | 301 | gi\|335331566 | GO! |
| 74 | gi\|75219081\|gid\|154\|Putative LMM glutenin 1 | Triticum aestivum | 41.23% | 54of444 | 1e-006 | 30.10% | 173 | gi\|75219081 | GO! |
| 75 | gi\|1137166044\|gid\|151\|Allergen alpha-gliadin, partial [Tr | Triticum monococcum subsp. aegilopoides | 40.04% | 30of444 | 0.00012 | 31.00% | 174 | gi\|1137166044 | GO! |
| 76 | gi\|21926\|gid\|154\|Putative glutenin-like protein product [ | Triticum turgidum subsp. durum | 40.03% | 46of444 | 6.1e-006 | 46.40% | 69 | gi\|21926 | GO! |
| 77 | gi\|21773\|gid\|154\|Putative LMW glutenin-like protein produ | Triticum aestivum | 40.03% | 35of444 | 0.0014 | 52.50% | 61 | gi\|21773 | GO! |
| 78 | gi\|508732627\|gid\|154\|Putative low molecular weight gluten | Triticum aestivum | 40.03% | 35of444 | 0.0012 | 52.50% | 61 | gi\|508732627 | GO! |
| 79 | gi\|170734\|gid\|154\|Putative gamma gliadin B-III | Triticum aestivum | 40.03% | 35of444 | 0.001 | 52.50% | 61 | gi\|170734 | GO! |
| 80 | gi\|21783\|gid\|154\|Putative LMW glutenin-like protein produ | Triticum aestivum | 40.02% | 45of444 | 7e-005 | 55.20% | 58 | gi\|21783 | GO! |
| 81 | gi\|897811\|gid\|160\|Allergen HMW gluten-like protein produc | Triticum aestivum | 40.00% | 58of444 | 1.1e-006 | 50.00% | 64 | gi\|897811 | GO! |
| 82 | gi\|21757\|gid\|151\|Allergen Gliadin-like protein product [T | Triticum aestivum | 39.99% | 10of444 | 0.00051 | 30.40% | 171 | gi\|21757 | GO! |
| 83 | gi\|75317968\|gid\|154\|Putative LMM glutenin 3 | Triticum aestivum | 39.97% | 48of444 | 1.1e-005 | 57.10% | 56 | gi\|75317968 | GO! |
| 84 | gi\|170718\|gid\|151\|Allergen alpha/beta-gliadin precursor | Triticum aestivum | 39.97% | 32of444 | 8.2e-005 | 43.20% | 74 | gi\|170718 | GO! |
| 85 | gi\|21765\|gid\|151\|Allergen Gliadin-like protein product [T | Triticum aestivum | 39.97% | 32of444 | 8.2e-005 | 43.20% | 74 | gi\|21765 | GO! |
| 86 | gi\|21673\|gid\|151\|Allergen Alpha/beta gliadin-like protein | Triticum aestivum | 38.76% | 23of444 | 0.00024 | 28.20% | 206 | gi\|21673 | GO! |
| 87 | gi\|170712\|gid\|151\|Allergen pre-alpha-/beta-gliadin A-II | Triticum aestivum | 38.10% | 8of444 | 0.0039 | 24.40% | 234 | gi\|170712 | GO! |

| | | | | | | | | | |
|---|---|---|---|---|---|---|---|---|---|
| 88 | gi\|170726\|gid\|151\|Allergen pre-alpha-/beta-gliadin A-III | Triticum aestivum | 38.10% | 13of444 | 0.029 | 26.80% | 213 | gi\|170726 | GO! |
| 89 | gi\|21761\|gid\|151\|Allergen Gliadin-like protein product [T | Triticum aestivum | 37.54% | 38of444 | 0.00092 | 31.40% | 169 | gi\|21761 | GO! |
| 90 | gi\|170722\|gid\|151\|Allergen pre-alpha-/beta-gliadin A-I | Triticum aestivum | 37.54% | 24of444 | 0.00082 | 29.90% | 167 | gi\|170722 | GO! |
| 91 | gi\|886967\|gid\|154\|Putative low molecular weight glutenin | Triticum aestivum | 37.53% | 10of444 | 0.021 | 27.00% | 189 | gi\|886967 | GO! |
| 92 | gi\|886965\|gid\|154\|Putative low molecular weight glutenin | Triticum aestivum | 37.52% | 16of444 | 0.042 | 28.10% | 178 | gi\|886965 | GO! |
| 93 | gi\|21755\|gid\|151\|Allergen Alpha/beta gliadin-like protein | Triticum aestivum | 37.50% | 10of444 | 0.00022 | 24.40% | 225 | gi\|21755 | GO! |
| 94 | gi\|170720\|gid\|151\|Allergen alpha/beta-gliadin precursor [ | Triticum aestivum | 37.50% | 10of444 | 0.00049 | 26.30% | 224 | gi\|170720 | GO! |
| 95 | gi\|886963\|gid\|154\|Putative low molecular weight glutenin | Triticum aestivum | 36.25% | 27of444 | 0.019 | 28.70% | 150 | gi\|886963 | GO! |
| 96 | gi\|62484809\|gid\|152\|Allergen putative gamma-gliadin [Trit | Triticum aestivum | 35.02% | 1of444 | 6.3 | 40.00% | 70 | gi\|62484809 | GO! |

| C3S7F1: Oleosin S2-2 [Brassica napus (Rape)] | |
|---|---|
| **Database** | AllergenOnline Database v19 (February 10, 2019) |
| **Input Query** | >query<br>MATVERRVQVDPTDKRIHLQPQYEGDVGYGYGYGGRADYKSSGPSSNQIVALIVGVPVGG<br>SLLALAGLTLAGSVIGLMLSVPLFLLFSPVIVPAAITIGLAVTAILASGLFGLTGLSSVS<br>WVLNYLRGTSDTVPEQLDYAKRRMADAVGYAGQKGKEMGQYVQDKAHEAHDTSLTTETTE<br>PGKTRRHT |
| **Length** | 188 |
| **Number of 80 mers** | 109 |
| **Number of Sequences with hits** | 10 |

| | Defline | Species | | | Full Alignment | Links |
|---|---|---|---|---|---|---|

| Hit # | | | Best %ID | # Hits > 35% | E-val | %ID | length | NCBI | Details |
|---|---|---|---|---|---|---|---|---|---|
| 1 | gi\|49617323\|gid\|2298\|Allergen oleosin [Corylus avellana] | Corylus avellana | 70.01% | 109of109 | 2.2e-042 | 52.50% | 158 | gi\|49617323 | GO! |
| 2 | gi\|113200509\|gid\|2283\|Putative oleosin 1 [Arachis hypogae | Arachis hypogaea | 64.98% | 104of109 | 6.5e-039 | 46.20% | 171 | gi\|113200509 | GO! |
| 3 | gi\|52001239\|gid\|2283\|Putative oleosin 2, partial [Arachis | Arachis hypogaea | 64.98% | 106of109 | 2.6e-038 | 52.30% | 149 | gi\|52001239 | GO! |
| 4 | gi\|10834827\|gid\|1893\|Putative oleosin [Sesamum indicum] | Sesamum indicum | 62.50% | 103of109 | 2.2e-031 | 45.90% | 157 | gi\|10834827 | GO! |
| 5 | gi\|71040655\|gid\|2284\|Putative oleosin 1 [Arachis hypogaea | Arachis hypogaea | 55.00% | 74of109 | 1e-021 | 43.80% | 128 | gi\|71040655 | GO! |
| 6 | gi\|122218540\|gid\|2284\|Putative Oleosin 2 | Arachis hypogaea | 53.80% | 71of109 | 4e-021 | 39.90% | 138 | gi\|122218540 | GO! |
| 7 | gi\|198250343\|gid\|1238\|Putative main allergen 15 kDa oleos | Sesamum indicum | 53.80% | 78of109 | 1.1e-021 | 40.90% | 132 | gi\|198250343 | GO! |
| 8 | gi\|5381321\|gid\|1238\|Putative 15 kDa oleosin [Sesamum indi | Sesamum indicum | 53.80% | 78of109 | 1.6e-021 | 40.90% | 132 | gi\|5381321 | GO! |
| 9 | gi\|29170509\|gid\|389\|Putative oleosin [Corylus avellana] | Corylus avellana | 50.00% | 68of109 | 1.1e-021 | 41.00% | 117 | gi\|29170509 | GO! |
| 10 | gi\|52001241\|gid\|2285\|Allergen oleosin 3 [Arachis hypogaea | Arachis hypogaea | 45.10% | 77of109 | 9.1e-021 | 38.20% | 144 | gi\|52001241 | GO! |

| C3S7F8: Oleosin S3-1 [Brassica napus (Rape] | |
|---|---|
| Database | AllergenOnline Database v19 (February 10, 2019) |
| Input Query | >query<br>MADTARTHHDITSRDQYPILGRDRDQYPYGRSDYQTSGQDYSKTRQIAKAATAVTAGGSL<br>LVLSSLTLVGTVIALTVATTLLVIFSPILVPALITVALLITGFLSSGGFGIADITVFSWI<br>YKYATGEHPQGSDKLDSARMKLGTKAQDIKDRAQYYGQQHTGGEHDRDRTRGTHHTTTTT |
| Length | 180 |
| Number of 80 mers | 101 |
| Number of Sequences with hits | 10 |

| Hit # | Defline | Species | Best %ID | # Hits > 35% | Full Alignment | | | Links | |
|---|---|---|---|---|---|---|---|---|---|
| | | | | | E-val | %ID | length | NCBI | Details |
| 1 | gi\|5381321\|gid\|1238\|Putative 15 kDa oleosin [Sesamum indi | Sesamum indicum | 76.20% | 101of101 | 6e-031 | 63.20% | 125 | gi\|5381321 | GO! |
| 2 | gi\|198250343\|gid\|1238\|Putative main allergen 15 kDa oleos | Sesamum indicum | 76.20% | 101of101 | 6e-031 | 63.20% | 125 | gi\|198250343 | GO! |
| 3 | gi\|29170509\|gid\|389\|Putative oleosin [Corylus avellana] | Corylus avellana | 73.80% | 98of101 | 1.1e-032 | 66.90% | 121 | gi\|29170509 | GO! |
| 4 | gi\|71040655\|gid\|2284\|Putative oleosin 1 [Arachis hypogaea | Arachis hypogaea | 70.00% | 94of101 | 1.2e-028 | 60.80% | 125 | gi\|71040655 | GO! |

| | | | | | | | | | | |
|---|---|---|---|---|---|---|---|---|---|---|
| 5 | gi\|122218540\|gid\|2284\|Putative Oleosin 2 | Arachis hypogaea | 68.80% | 94of101 | 4.6e-029 | 61.60% | 125 | gi\|122218540 | GO! |
| 6 | gi\|52001241\|gid\|2285\|Allergen oleosin 3 [Arachis hypogaea | Arachis hypogaea | 65.00% | 97of101 | 5.3e-024 | 48.70% | 156 | gi\|52001241 | GO! |
| 7 | gi\|10834827\|gid\|1893\|Putative oleosin [Sesamum indicum] | Sesamum indicum | 48.10% | 79of101 | 6.1e-015 | 36.30% | 171 | gi\|10834827 | GO! |
| 8 | gi\|49617323\|gid\|2298\|Allergen oleosin [Corylus avellana] | Corylus avellana | 45.70% | 55of101 | 1.2e-013 | 31.50% | 149 | gi\|49617323 | GO! |
| 9 | gi\|113200509\|gid\|2283\|Putative oleosin 1 [Arachis hypogae | Arachis hypogaea | 41.29% | 54of101 | 1.3e-015 | 32.40% | 148 | gi\|113200509 | GO! |
| 10 | gi\|52001239\|gid\|2283\|Putative oleosin 2, partial [Arachis | Arachis hypogaea | 41.29% | 55of101 | 1.9e-014 | 33.30% | 129 | gi\|52001239 | GO! |

---

---

| **Q6W7E8: Enolase [Brassica campestris (Field mustard)]** | |
|---|---|
| **Database** | AllergenOnline Database v19 (February 10, 2019) |
| **Input Query** | >query<br>MATITAVKARQIFDSRGNPTVEVDVHTSSGVKVTAAVPSGASTGIYEALELRDGGSDYLG<br>KGVSKAVGNVNSIIGPASIGKDPTQQTAIDNFMVHELDGTQNEWGWCKQKLGANAILAVS<br>LAVCKAGAVVSGIPLYKHIANLAGNPKIVLPVPAFNVINGGSHAGNKLAMQEFMILPVGA<br>SSFKEAMKMGVEVYHNLKSVIKKKYGQDATNVGDEGGFAPNIQENKEGLELLKTAIEKAG<br>YTGKVVIGMDVAASEFYSSDKTYDLNFKEENNNGSQKISGDALKDLYKSFVAEYPIVSIE<br>DPFDQDDWEHYAKMTAECGDNVQIVGDDLLVTNPKGVAKAIAEKSCNALLLKVNQIGSVT<br>ESIEAVKMSKRAGWGVMASHRSGETEDTFIADLSVGLSTGQIKTGAPCRSERLAKYNQLL<br>RIEEELGSEAVYAGANFRKPVEPY |
| **Length** | 444 |
| **Number of 80 mers** | 365 |
| **Number of Sequences with hits** | 16 |

| Hit # | Define | Species | Best %ID | # Hits > 35% | Full Alignment | | | Links | |
|---|---|---|---|---|---|---|---|---|---|
| | | | | | E-val | %ID | length | NCBI | Details |
| 1 | gi\|14423687\|gid\|586\|Putative Enolase 2 (2-phosphoglycerat | Hevea brasiliensis | 98.80% | 365of365 | 1.1e-170 | 89.60% | 442 | gi\|14423687 | GO! |
| 2 | gi\|9581744\|gid\|586\|Putative enolase, isoform 1 [Hevea bra | Hevea brasiliensis | 98.80% | 365of365 | 1.6e-171 | 90.00% | 442 | gi\|9581744 | GO! |

| | | | | | | | | | |
|---|---|---|---|---|---|---|---|---|---|
| 3 | gi\|576011129\|gid\|1955\|Allergen RecName: Full=Alpha-enolas | Thunnus albacares | 88.70% | 365of365 | 2.9e-129 | 69.50% | 442 | gi\|576011129 | GO! |
| 4 | gi\|385145180\|gid\|1959\|Allergen enolase [Salmo salar] | Salmo salar | 88.70% | 365of365 | 5.1e-130 | 69.90% | 442 | gi\|385145180 | GO! |
| 5 | gi\|197632415\|gid\|1959\|Allergen enolase 3-2 [Salmo salar] | Salmo salar | 85.02% | 365of365 | 7.2e-125 | 68.00% | 441 | gi\|197632415 | GO! |
| 6 | gi\|46048765\|gid\|2710\|Allergen beta-enolase [Gallus gallus | Gallus gallus | 83.80% | 365of365 | 3.6e-119 | 65.10% | 441 | gi\|46048765 | GO! |
| 7 | gi\|232054\|gid\|396\|Allergen Enolase 1 (2-phosphoglycerate | Candida albicans | 82.50% | 365of365 | 1.5e-111 | 63.10% | 444 | gi\|232054 | GO! |
| 8 | gi\|30314940\|gid\|103\|Putative enolase [Rhodotorula mucilag | Rhodotorula mucilaginosa | 80.00% | 365of365 | 9.5e-107 | 61.00% | 441 | gi\|30314940 | GO! |
| 9 | gi\|6015094\|gid\|491\|Putative Enolase (2-phosphoglycerate d | Davidiella tassiana | 77.50% | 365of365 | 1.6e-108 | 60.70% | 443 | gi\|6015094 | GO! |
| 10 | gi\|83288046\|gid\|329\|Putative Enolase (2-phosphoglycerate | Aspergillus fumigatus | 77.50% | 365of365 | 7.6e-108 | 61.60% | 443 | gi\|83288046 | GO! |
| 11 | gi\|467660\|gid\|491\|Putative enolase; phosphopyruvate hydra | Davidiella tassiana | 77.50% | 365of365 | 1.8e-106 | 59.80% | 443 | gi\|467660 | GO! |
| 12 | gi\|13925873\|gid\|329\|Putative enolase [Aspergillus fumigat | Aspergillus fumigatus | 77.50% | 365of365 | 1.2e-107 | 61.40% | 443 | gi\|13925873 | GO! |
| 13 | gi\|14423684\|gid\|66\|Putative Enolase (2-phosphoglycerate d | Alternaria alternata | 76.20% | 365of365 | 2.4e-106 | 60.60% | 444 | gi\|14423684 | GO! |
| 14 | gi\|13991101\|gid\|249\|Putative enolase [Penicillium citrinu | Penicillium citrinum | 76.20% | 365of365 | 6.7e-105 | 60.50% | 443 | gi\|13991101 | GO! |
| 15 | gi\|14585753\|gid\|1031\|Putative enolase [Curvularia lunata] | Cochliobolus lunatus | 75.00% | 365of365 | 4.2e-098 | 56.50% | 448 | gi\|14585753 | GO! |
| 16 | gi\|344049993\|gid\|2295\|Putative enolase, partial [Fennerop | Fenneropenaeus merguiensis | 68.80% | 114of365 | 3.7e-031 | 66.70% | 117 | gi\|344049993 | GO! |

| **Q42618- BRANA Beta-glucosidase [Brassica napus (Rape)]** | |
|---|---|
| **Database** | AllergenOnline Database v19 (February 10, 2019) |

| | |
|---|---|
| **Input Query** | >query<br>MKFPLLGLLLLVTLVGSPTRAEEGPVCPKTETLSRASFPEGFMFGTATASYQVEGAVNEG<br>CRGPSLWDIYTKKFPHRVKNHNADVAVDFYHRFREDIKLMKKLNTDALRLSIAWPRIFPH<br>GRMEKGNSKEGVQFYHDLIDELLKNDLTPLVTIFHWDMPADLEDEYGGFLSERVVPDFVE<br>YANFTFHEYGDKVKNWITFNEPWVFSRSAYDVGKKAPGRCSPYIKDFGHLCQDGRSGFEA<br>YVVSHNLLVSHAEAVDAFRKCEKCKGDKIGIAHSPAWFEPEDVEGGQRTVDRVLDFIMGW<br>HLDPTTYGDYPQSMKDAVGARLPKFTKAQKAKLKGSADFVGINYYSSFYAKASEKPDYRQ<br>PSWATDSLVEFEPKTVDGSVKIGSQPSTAKMAVYAAGLRKLVKYIKDRYGNPEIIITENG<br>YGEDLGEKDTDHSVALNDHNRKYYHQRHLLSLHQAICEDKVNVTSYFVWSLMDNFEWLDG<br>YTARFGLYYIDFQNNLTRMEKESATCSLNSSNRA |
| **Length** | 514 |
| **Number of 80 mers** | 435 |
| **Number of Sequences with hits** | 0 |

No Matches of Greater than 35% Identity Found

| **O23733-RAJU Cysteine synthase [Brassica juncea]** | |
|---|---|
| **Database** | AllergenOnline Database v19 (February 10, 2019) |
| **Input Query** | >query<br>MASRIAKDVTELIGNTPLVYLNNVAEGCVGRVAAKLEMMEPCSSVKDRIGFSMISDAEQK<br>GLIKPGESVLIEPTSGNTGVGLAFTAAAKGYKLIITMPASMSVERRIILLAFGVELVLTD<br>PAKGMKGAIAKAEEILAKTPNGYMLQQFENPANPKIHYETTGPEIWKGTDGKIDGFVSGI<br>GTGGTITGAGKYLKEQNPNVKLYGVEPIESAILSGGKPGPHKIQGIGAGFIPSVLEVDLI<br>DEVVQVSSDESIDMARLLALKEGLLVGISSGAAAAAAIKLAKRPENAGKLFVAVFPSFGE<br>RYLSTVLFDATRKEAESMTFQA |
| **Length** | 322 |
| **Number of 80 mers** | 243 |

| Number of Sequences with hits | 1 |
|---|---|

| Hit # | Defline | Species | Best %ID | # Hits > 35% | Full Alignment | | | Links | |
|---|---|---|---|---|---|---|---|---|---|
| | | | | | E-val | %ID | length | NCBI | Details |
| 1 | gi\|729764\|gid\|519\|Putative Heat shock 70 kDa protein (All | Davidiella tassiana | 35.40% | 13of243 | 1.2 | 30.70% | 140 | gi\|729764 | GO! |

| **C3S7H5-BRANA Caleosin CLO1-2 [Brassica napus (Rape)]** | |
|---|---|
| **Database** | AllergenOnline Database v19 (February 10, 2019) |
| **Input Query** | >query<br>MSTATEIMGRDAMATVAPYAPVTFHRRARVDMDDRLPKPYMPRALQAPDREHPYGTPGHK<br>NYGLSVLQQHVAFFDLYDNGIIYPWETYSGLRMLGFNIIVSLIAAAVINLALSYATLTGW<br>FPSPFFPIYIHNIHKSKHGSDSRTYDNEGRFMPVNLELIFSKYAKTLPDKLSLGELWEMT<br>QGQRDAWDIFGWFASKIEWGLLYLLARDEEGFLSKEAIRRCFDGSLFEYCAKIYAGINED<br>KTAYY |
| **Length** | 245 |
| **Number of 80 mers** | 166 |
| **Number of Sequences with hits** | 0 |

No Matches of Greater than 35% Identity Found

| **P13244- Malate synthase. glyoxysomal [Brassica napus (Rape)]** | |
|---|---|
| **Database** | AllergenOnline Database v19 (February 10, 2019) |

| | |
|---|---|
| **Input Query** | >query<br>MELETSVYRPNVAVYDSPDGVEVRGRYDQVFAKILTRDALGFVAELQREFRGHVRYAMEC<br>RREVKRRYNSGAVPGFDPSTKFIRDGEWVCASVPPAVADRRVEITGPVERKMIINALNSG<br>AKVFMADFEDALSPSWENLMRGQVNLKDAVDGSITFNDKARNKVYKLNDQVAKLFVRPRG<br>WHLPEAHILIDGEPATGCLVDFGLYFFHNYAKFRQTQGSGFGPFFYLPKMEHSREAKIWN<br>SVFERAEKMAGIERGSIRATVLIETLPAVFQMNEILYELRDHSVGLNCGRWDYIFSYVKT<br>FQAHPDRLLPDRVLVGMGQHFMRSYSDLLIRTCHKRGVHAMGGMAAQIPIRDDPKANEMA<br>LDLVKKDKLREVRAGHDGTWAAHPGLIPICMDAFSHMGNNPNQIKSMKRDDASAITEEDL<br>LQIPRGVRTLEGLRLNTRVGIQYLAAWLTGSGSVPLYNLMEDAATAEISRVQNWQWIRYG<br>VELDGDGLGVRVSKELFGRVVEEEMERIEKEVGKDKFKRGMYKEACKMFTKQCTAAELDD<br>FLTLAVYDHIVAHYPINASRL |
| **Length** | 561 |
| **Number of 80 mers** | 482 |
| **Number of Sequences with hits** | 0 |

No Matches of Greater than 35% Identity Found

| **Q7XZT2-BRAJU Glutathione S-transferase 3 [Brassica juncea ]** | |
|---|---|
| **Database** | AllergenOnline Database v19 (February 10, 2019) |
| **Input Query** | >query<br>MAGIKVFGSAASPSTRRVLLALHEKNLDFELVNVELKDGEHKKEPFLSRNPFGKVPAFED<br>GDLKLFESRAITQYIAHRYEGQGTNLLPADSKNIAHYAIMAIGLEVEAHQFDPVASKLAW<br>EQVFKNFYGLTTDQAVVAEEEAKLAKVLDVYEARLKEFKYLAGETFTLTDLHHIPVIQYL<br>LGTPTKKLFTERPRVNEWVAEITKRPASQKILQ |

| | |
|---|---|
| **Length** | 213 |
| **Number of 80 mers** | 134 |
| **Number of Sequences with hits** | 4 |

| Hit # | Defline | Species | Best %ID | # Hits > 35% | Full Alignment | | | Links | |
|---|---|---|---|---|---|---|---|---|---|
| | | | | | E-val | %ID | length | NCBI | Details |
| 1 | gi\|359326557\|gid\|856\|Allergen glutathione S transferase c | Periplaneta americana | 40.00% | 14of134 | 1.2e-006 | 29.40% | 163 | gi\|359326557 | GO! |
| 2 | gi\|1337340498\|gid\|856\|Allergen glutathione S transferase | Periplaneta americana | 40.00% | 14of134 | 1.2e-006 | 29.20% | 161 | gi\|1337340498 | GO! |
| 3 | gi\|161137518\|gid\|1353\|Putative delta class glutathione S- | Blattella germanica | 38.80% | 15of134 | 4.9e-006 | 29.40% | 197 | gi\|161137518 | GO! |
| 4 | gi\|60678789\|gid\|856\|Allergen Per a 5 allergen [Periplanet | Periplaneta americana | 38.80% | 13of134 | 1.5e-005 | 28.80% | 163 | gi\|60678789 | GO! |
